# Cerebral Biochemical Pathways in Experimental Autoimmune Encephalomyelitis and Adjuvant Arthritis: A Comparative Metabolomic Study

**DOI:** 10.1371/journal.pone.0056101

**Published:** 2013-02-14

**Authors:** Norbert W. Lutz, Carla Fernandez, Jean-François Pellissier, Patrick J. Cozzone, Evelyne Béraud

**Affiliations:** 1 Center for Biological and Medical Magnetic Resonance (CRMBM), National Center for Scientific Research (Joint Research Unit 7339), Aix-Marseille University, Marseille, France; 2 Biological Oncology and Oncopharmacology Research Center (CRO2), National Institute of Health and Medical Research (Joint Research Unit 911), Aix-Marseille University, Marseille, France; Weizmann Institute of Science, Israel

## Abstract

Many diseases, including brain disorders, are associated with perturbations of tissue metabolism. However, an often overlooked issue is the impact that inflammations outside the brain may have on brain metabolism. Our main goal was to study similarities and differences between brain metabolite profiles of animals suffering from experimental autoimmune encephalomyelitis (EAE) and adjuvant arthritis (AA) in Lewis rat models. Our principal objective was the determination of molecular protagonists involved in the metabolism underlying these diseases. EAE was induced by intraplantar injection of complete Freund’s adjuvant (CFA) and spinal-cord homogenate (SC-H), whereas AA was induced by CFA only. Naive rats served as controls (n = 9 for each group). Two weeks after inoculation, animals were sacrificed, and brains were removed and processed for metabolomic analysis by NMR spectroscopy or for immunohistochemistry. Interestingly, both inflammatory diseases caused similar, though not identical, changes in metabolites involved in regulation of brain cell size and membrane production: among the osmolytes, taurine and the neuronal marker, *N*-acetylaspartate, were decreased, and the astrocyte marker, *myo*-inositol, slightly increased in both inoculated groups compared with controls. Also ethanolamine-containing phospholipids, sources of inflammatory agents, and several glycolytic metabolites were increased in both inoculated groups. By contrast, the amino acids, aspartate and isoleucine, were less concentrated in CFA/SC-H and control vs. CFA rats. Our results suggest that inflammatory brain metabolite profiles may indicate the existence of either cerebral (EAE) or extra-cerebral (AA) inflammation. These inflammatory processes may act through distinct pathways that converge toward similar brain metabolic profiles. Our findings open new avenues for future studies aimed at demonstrating whether brain metabolic effects provoked by AA are pain/stress-mediated and/or due to the presence of systemic proinflammatory molecules. Regardless of the nature of these mechanisms, our findings may be of interest for future clinical studies, e.g. by *in-vivo* magnetic resonance spectroscopy.

## Introduction

Experimental autoimmune encephalomyelitis (EAE) exhibits many of the features that characterize the human chronic inflammatory brain disease, multiple sclerosis (MS): immune cell infiltration, demyelination (and remyelination), oligodendrocyte damage and axonal loss, as well as microvascular abnormalities and impairment of neural metabolism [1]. One of the aims of EAE research is the understanding of effector pathogenic mechanisms. While EAE is not an ideal model of MS, it is certainly useful for pinpointing some basic characteristics that may serve to design MS treatments.

EAE can be induced either actively by immunization of susceptible animals with encephalitogenic myelin antigens emulsified in complete Freund’s adjuvant (CFA) or, since EAE is a T cell-mediated disease [2], [3], passively by adoptive transfer of T syngeneic lymphocytes specific to a myelin antigen. The extent of demyelination varies according to species, antigens and sensitization regimens of the animals. Chronic relapsing EAE can be induced in Lewis rats by immunization with spinal cord homogenate (SC-H) emulsified in CFA. Injection of CFA, a water-in-mineral-oil emulsion containing killed mycobacteria, causes a slow release of antigens at the site of inoculation. Also, mycobacteria display antigenic patterns, which are recognized by innate immune cells (antigen presenting cellssuch as dendritic cells and macrophages) expressing anti-mycobacterial molecules such as nitric oxide synthase as well as cytokines including interleukin 1β (Il-1β) and tumor necrosis factor α (TNF-α); see [4] for a recent review). CFA is a well-established immunopotentiator that induces and enhances the innate and adaptive immune responses. For this reason CFA is employed to facilitate active induction of EAE in laboratory animals by increasing incidence and frequency of immune responses, and by decreasing delays of EAE appearance.

Several pathways have been explored to highlight physiological and biochemical processes underlying the action of CFA in EAE induction [5]. However, the responsible mechanisms remain largely unresolved, and their elucidation is complicated by the fact that CFA injection *per se* causes chronic inflammation of the joints, pain and hyperalgesia, an experimental disease named adjuvant arthritis (AA), which is also T-cell mediated. AA serves as a model of rheumatoid arthritis [6] and of chronic inflammatory pain [7]. Of special note, AA and EAE do not concomitantly occur in animals sensitized to CFA/SC-H; EAE is dominant (own observations; data not shown).

Peripheral injections of CFA provoke leakage of serum proteins through the blood-brain barrier (BBB) [8]. Namer et al [9] demonstrated that antibodies directed against mannan of the *Mycobacterium tuberculosis* (Mt) envelope could cause BBB breakdown, while Huber et al [10] showed that inflammation produced by subcutaneous injection of CFA alters BBB permeability by inducing reorganization of tight junctions. Rabchevsky reported that BBB alteration occurs without inducing reactive gliosis [8]. Others found that glial activation [11], [12], [13] and cyclooxygenase-2 induction [14] are possibly linked to an increase of proinflammatory cytokines (with IL-1β as a prime candidate) originating from resident cells of the central nervous system (CNS) rather than from peripheral sites [11], [14], [15]. Whether a BBB breakdown that leads to reactive gliosis is accompanied by the entry of inflammatory cells and components into the CNS is still an open question. In the same line, how nerve inflammation leads to glial activation remains unclear; at least three mechanisms have been proposed [16]. Regardless of how ultimately glia become activated, new approaches are necessary to better define the molecular protagonists involved in the underlying mechanisms.

Metabolomics, the systematic study of tissue and biofluid metabolites without prior selection for specific pathways, has emerged as a new comprehensive approach in the investigation of biological processes. Metabolomics is a “hypothesis-generating” rather than a “hypothesis-driven” method, akin to genomics and proteomics. While the latter two have enjoyed significant application in MS and EAE research [17], metabolomic studies are still sparse in MS [18], [19], [20], and are very rare in EAE [21], [22]. In this study we use metabolomic nuclear magnetic resonance (NMR) spectroscopy of brain extracts to assess biochemical processes underlying (i) EAE caused by the injection of an emulsion of spinal cord homogenate in CFA (CFA/SC-H), and (ii) AA with significant peri-articular inflammation, caused by the injection of CFA alone. To the best of our knowledge, this is the first characterization of metabolic processes underlying the action of CFA *vs*. CFA/SC-H, in conjunction with CNS histopathology and immunohistochemistry (IHC), and the first metabolomic EAE and AA study based on extracts of both water-soluble metabolites and phospholipids of brain tissue.

## Materials and Methods

### 1. Ethics Statement/Animals

Animal studies on rats followed the guidelines valid in France, and were approved by the local Ethics Committee (n°40.04, University of Aix-Marseille Medical School, Marseille, France). Female Lewis rats (12–13 weeks old) were obtained from Janvier Breeding Center (Le Genet ST Isle, France) and kept in the animal facilities of the Centre de Formation et de Recherches Expérimentales Médico-Chirurgicales (University of Aix-Marseille Medical School). Pre-established clinical criteria in determining a need for euthanization of animals to prevent unnecessary suffering were: (a) animals presenting a score of 6 (complete hind limb paralysis, incontinence, without eating or drinking); and (b), regardless of EAE score: animals with skin damage from abrasion or urine scald, animals that do not eat or drink, animals with more than a few microliters of hemorrhage, vocalization, self mutilation, wounds, atypical neurologic signs such as epilepsia, impairment of breathing, lost of consciousness, imbalance or prostration, evident signs of infection at the sites of immunization, retention of urine, and weight of loss ≥25%. We certify that during our study, none of the rats reached any of the criteria requiring euthanization.

Guinea pig spinal cord (SC) tissue was purchased from Harlan Laboratories, Shardlow, UK; Harlan ensures that all national and international rules, regulations, standards, and guidelines are met (http://www.harlan.com/about_harlan_laboratories/animal_welfare.hl).

### 2. EAE and AA Induction

Guinea pig SC tissue was used after storage at −80°C. One gram Guinea pig SC and 1 mL saline were homogenized in a blender (Virtis, Gardiner, NY, USA). The SC homogenate (SC-H) was then emulsified in 2 mL of incomplete Freund’s adjuvant (Sigma-Aldrich, Saint Quentin, Fallavier, France) supplemented with 20 mg of Mt H37Ra (Difco Laboratories, Detroit, MI, USA), as described previously with slight modifications [23]. One group of Lewis rats (n = 9) received 0.1 mL of the CFA/SC-H emulsion by intraplantar injection (EAE group). A second group (n = 9) received 0.1 mL of an emulsion containing CFA (500 µg Mt) without SC-H, under the same conditions (AA group). A third group (n = 9) was not injected and served as a control group (Contr).

### 3. Clinical Assessment of EAE and Selection of Animals

Immunized Lewis rats were observed daily to follow the clinical course of EAE, and were scored as follows: 0 = normal, 1 = loss of tail tonicity, 2 = weakness of one or both hind legs, or mild ataxia, 3 = severe ataxia or paralysis of both hind legs, 4 = severe paralysis with urinary incontinence, 5 = quadriplegia or moribund state. A part of the EAE Lewis rats (n = 6) were sacrificed and used for metabolomic NMR spectroscopy on the day they displayed clinical signs of EAE with score 4 (usually 12–15 days after immunization). On the same day, CFA and control rats were sacrificed for metabolomic analysis. The three remaining rats of the EAE group, randomly chosen for observation over a 50-day period, showed chronic-relapsing development of the disease.

### 4. Clinical Assessment of AA

The disease was monitored by observation of all four limbs in each animal, based on the degree of joint and peri-articular inflammation, redness, and deformity [24].

### 5. Sample Preparation for Metabolomic Study

Briefly, anesthetized animals were sacrificed, and the brain was removed, freeze-clamped, weighed, and extracted with methanol/chloroform/water. After phase separation, solvents were evaporated, and samples were redissolved in water (for analysis of water-soluble metabolites), or in methanol/chloroform/water containing a chelating agent (for analysis of phospholipids) [25], [26]. A detailed description of the protocol is provided in Section S1.1.

### 6. NMR Spectroscopy

^1^H and ^31^P NMR spectra at 400.1 and 162.0 MHz, respectively, were obtained on a 9.4 T AVANCE 400 wide-bore Fourier transform NMR spectrometer from Bruker (Wissembourg, France), and were analyzed with Bruker’s Topspin software (further technical details have been described elsewhere [27]). NMR signals were assigned based on previous work [27], and by spiking extracts with original compounds where necessary. Absolute metabolite concentrations were calculated as µmol/g brain tissue (wet weight), and relative metabolite concentrations were obtained as percent of total metabolite concentration (see Section S1.2. for more details).

### 7. Neurohistopathology and Immunohistochemistry

The brain (the entire organ, or one hemisphere) of the sacrificed animal was sectioned at the level of the midbrain and the hindbrain. The spinal cord was divided into cervical, thoracic, and lumbar segments. Paraffin sections (4 µm thick) of the CNS were stained with hematoxylin-eosin and Loyez stain to assess inflammatory cell infiltrates and demyelination, respectively. At least ten sections from each area, at different levels, were examined for each rat.

Sections were incubated with an antibody to glial fibrillary acidic protein (GFAP) (rabbit polyclonal anti-GFAP, diluted 1∶500; Biocare Medical, Concord, CA, USA) to label astrocytes and with an antibody to ionized calcium binding adaptor molecule (IBA1) (rabbit polyclonal anti-IBA1, diluted 1∶2000; Wako, Richmond, VA, USA). Rabbit-on-rodent polymer (Biocare Medical) was employed to label activated microglia. This polymer and biotinylated goat anti-rabbit immunoglobulin G (IgG) (Vector Laboratories, Burlingame, CA, USA) with the Vectastain ABC Elite Reagent (Vector Laboratories) were used to detect anti-GFAP and anti-IBA1, respectively (Seventh Wave Laboratories, Chesterfield, MO, USA). To identify infiltrating cells, sections were incubated with an antibody to CD3 (rabbit IgG) from Dako (Carpinteria, CA, USA) followed by biotinylated goat anti-rabbit IgG (Vector Laboratories) antibodies. Automated immunohistochemical detection of CD3 was performed using the Ventana Benchmark XT automate (Ventana Medical Systems SA, Illkirch, France).

DAB chromagen (Biocare Medical) was used for immunostains. The slides were lightly counter-stained with Mayer’s hematoxylin (Sigma-Aldrich). Additional slides were cut from normal and CFA brain and spinal cord blocks and negative control rabbit IgG (Biocare Medical; used for GFAP control) or normal rabbit fraction (DAKO; used for IBA1 control) was applied to these slides as negative controls for the immunostaining procedures. The slides were examined microscopically to evaluate the immunoreactivity. The intensity of chromagen staining in the positive cells was scored as 3 (moderate) or 4 (marked).

### 8. Statistics

The statistical methods employed to detect significant metabolic differences between groups included parametric and nonparametric tests. Furthermore, linear-trend analysis was applied to determine whether the metabolite concentration means increase (or decrease) systematically as the three groups were ordered in a particular sequence (Prism 5.0, GraphPad, San Diego, CA, USA). In addition, we used the measured metabolite concentrations in multivariate analyses with the aim to verify whether the rat brain metabolome can be employed to reliably separate and classify animals from the three groups studied in this work. To this end, we tested both an unsupervised and a supervised technique, i.e. principal component analysis (PCA) and linear discriminant analysis (LDA), respectively (JMP 9.0.0, SAS Institute, Cary, NC, USA). A comprehensive description of our statistical procedures is presented in Section S1.3.

## Results

Results were obtained using brains of rats with overt EAE (*i.e.,* suffering from paralysis of both hind legs) at day 12 to 15 post immunization. Rats injected with CFA alone developed AA beginning with an inflammation of the joints, followed by progressively increasing peri-articular inflammation. In the following sections, global metabolic trends are presented, followed by the evaluation of specific metabolite classes and individual metabolites. This metabolomic analysis is complemented by neurohistopathology and immunohistochemistry results of the CNS. Further results are presented in Text S1, Section S2, Tables S1, S2, S3, S4, S5, S6, and in Figures S1, S2, S3, S4.

### 1. General Metabolic Trends

**Figure 1:**
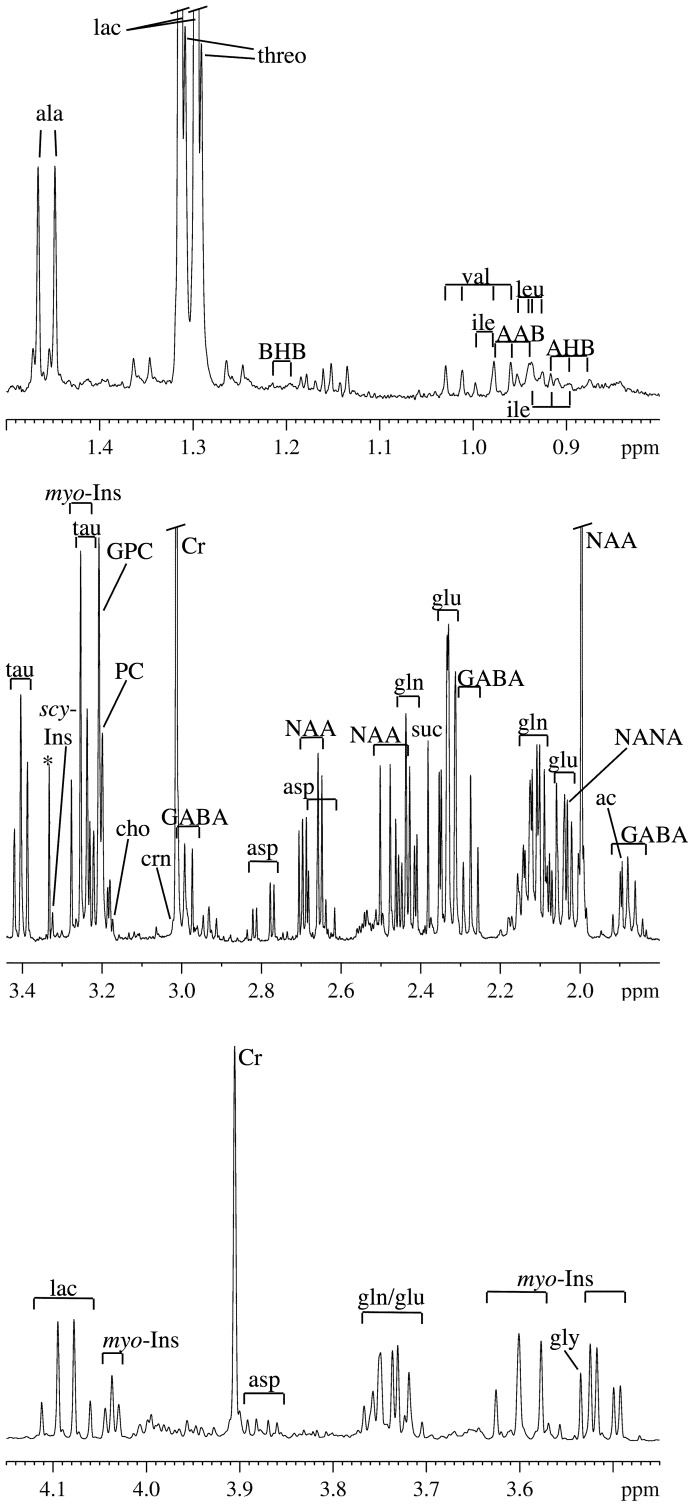
Subregion of a typical ^1^H NMR spectrum (400 MHz) of the aqueous phase of a brain tissue extract from a normal female Lewis rat. The asterisk denotes the methyl resonance due to a methanol impurity. For abbreviations see text and Table S4 D.

**Figure 2:**
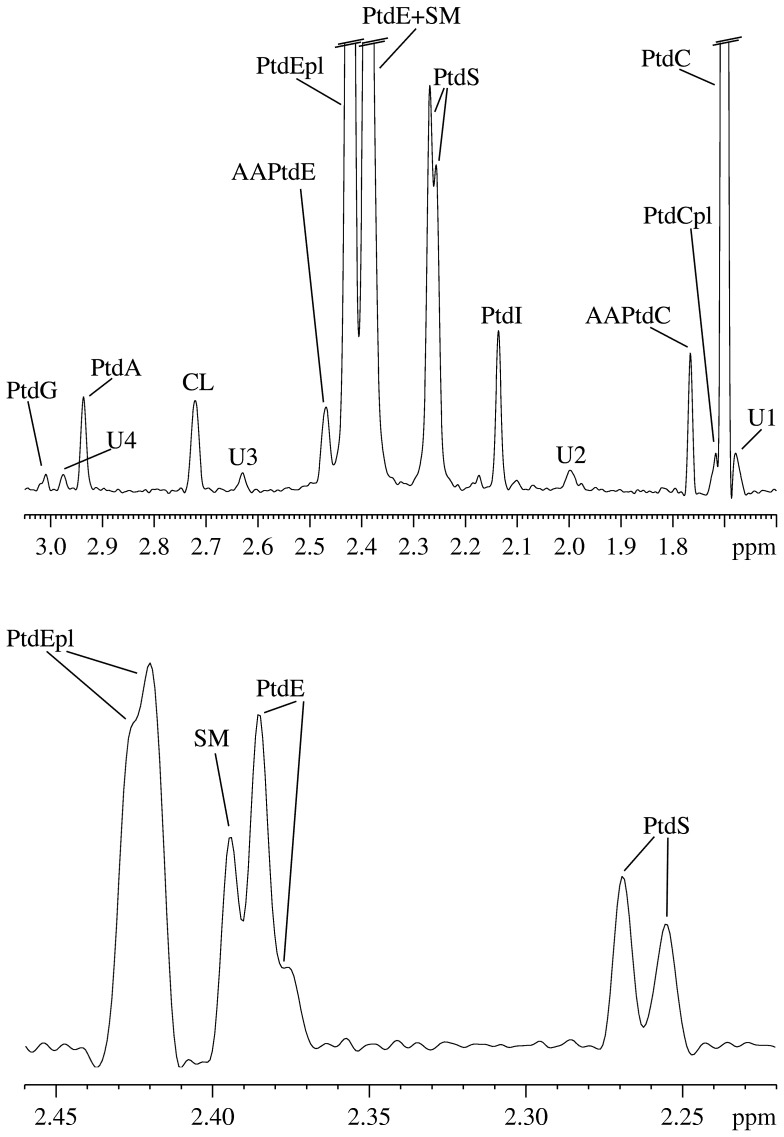
Subregion of a typical ^31^P NMR spectrum (162 MHz) of the organic phase of a brain tissue extract from a female Lewis rat; same brain tissue as for Fig. 1. For abbreviations see text and Table S1 D.

**Figure 3:**
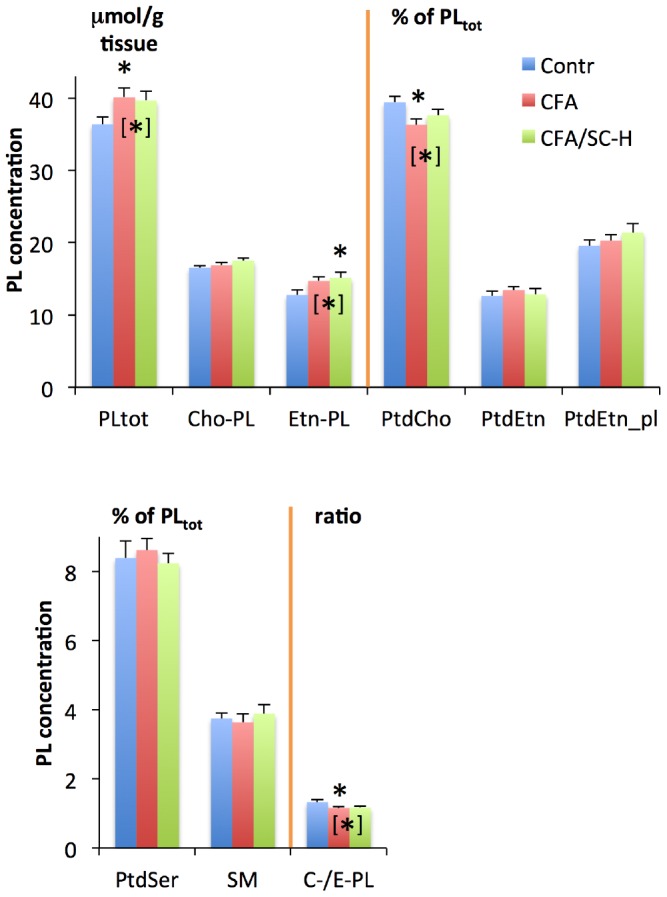
Absolute and relative concentrations of the most relevant rat brain PLs in control animals, in animals injected with CFA, and in animals showing clinical signs of EAE with score 4 after CFA/SC-H injection (means and standard errors). Unbracketed asterisks indicate statistically significant differences between the CFA or CFA/SC-H group on the one hand and the control group on the other, based on the tests given in Table 1. Bracketed asterisks indicate significant differences between the combined values of two groups and values of the remaining group, based on the tests given in Table S6 C. PtdEtn_pl = PtdEtn_plas_ (ethanolamine plasmalogen). Further PL concentrations and statistical results are provided in Figs. S1 and S3 and in Tables S1, S5 and S6, including also abbreviations (Table S1 D).

**Figure 4:**
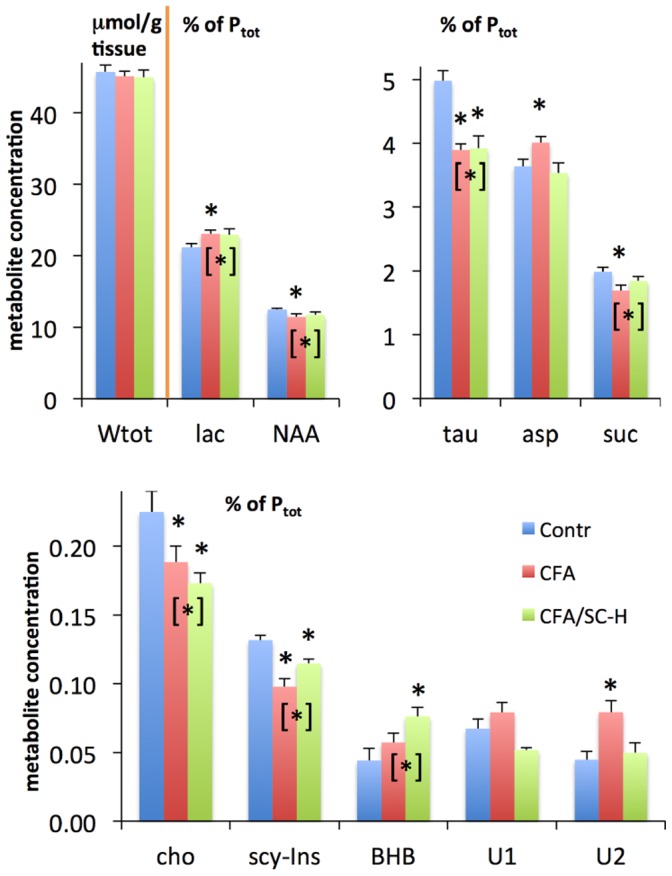
Absolute and relative concentrations of the most relevant water-soluble rat brain metabolites in control animals, animals injected with CFA, and animals showing clinical signs of EAE with score 4 after CFA/SC-H injection (means and standard errors). Unbracketed asterisks indicate statistically significant differences between the CFA or CFA/SC-H group on the one hand and the control group on the other, based on the tests given in Table 1. Bracketed asterisks indicate significant differences between the combined values of two groups and values of the remaining group, based on the tests given in Tables S3 C. Further metabolite concentrations and statistical results are provided in Figs. S2 and S4 and in Tables S3 to S4, including also abbreviations (Table S4 D).

The metabolites quantitated in this study fall into two classes that are located in distinct tissue environments prior to brain tissue extraction: (i) water-soluble metabolites, mostly consisting of amino acids, hydroxylated carboxylic acids, osmolytes and other small molecules that are predominantly found in various intracellular microenvironments, and, to a much lesser extent, in the interstitial space and in capillaries; and (ii) phospholipids (PLs), which are almost exclusively located in cell membranes. Characteristic ^1^H NMR spectra of water-soluble metabolites and ^31^P NMR spectra of PLs are shown in Figs. 1 and 2, respectively. An overview of the most striking quantitative results is presented in Figs. 3 and 4, and in Table 1 (see Tables S1 D and S4 D for lists of all quantitated metabolites).

**Table 1:** Significant differences in brain metabolite concentrations.

CFA vs. Contr	CFA/SC-H vs. Contr	CFA/SC-H vs. CFA
NAA ↓	cho ↓	U1 ↓
suc ↓	tau ↓	asp ↓
scy-Ins ↓	BHB ↑	*scy-Ins ↑
tau ↓	*scy-Ins↓	*U2 ↓
U2 ↑		*AAPtdEtn ↑
asp ↑		
*lac ↑		
*cho* ↓		
PtdCho ↓		
PtdIns_sum ↓		
GroPtdCho ↓		
Cho-PL ↓		
*C−/E-PL ↓		
*PL_tot_ ↑		

Pairwise comparisons between rats inoculated with CFA or CFA/SC-H, and control animals. Upward (downward) arrows indicate increased (decreased) relative concentrations for the first vs. the second group compared in each column, except for C−/E-PL and PL_tot_ (ratios and absolute concentrations, respectively). All differences shown in this table were statistically significant at the P<0.05 level in tests other than Newman-Keuls and Bonferroni, except for metabolites given in *italics* (P<0.05 in Newman-Keuls tests only), and preceded by an asterisk (P<0.1 in Bonferroni tests). For complete statistical results see Tables S1 to S6 and Section S1.3. Abbreviations: GroPtdCho, the sum of all PLs derived from PtdCho; PtdIns_sum, the sum of PtdIns and an associated PL, probably derived from PtdIns. For further abbreviations see text, and Tables S1 D and S4 D.

Total absolute PL concentrations (PL_tot_) expressed as µmol/g brain tissue (wet weight) were virtually identical in CFA and CFA/SC-H animals, and were increased in these groups by about 10% when compared to Contr (statistically significant for the CFA group, and for the CFA and CFA/SC-H groups combined, Fig. 3). Since PLs almost exclusively exist as membranous structures, a general PL increase has to be considered as an indicator of increased concentration of membrane material.

In contrast to PL_tot_, the total absolute concentration of water-soluble metabolites (W_tot_) was hardly changed in inoculated vs. control rats (insignificant decrease, P ≈ 0.24; Fig. 4). However, for 10 of 36 individual water-soluble metabolites, concentrations were found to be significantly different among the animal groups compared, whereas only 3 of 22 PL classes showed significant differences (absolute or relative concentrations), as did 4 PL-derived parameters of interest. Additional significant differences were detected when data from two groups were pooled and compared with the remaining group, or when linear trends were studied among all three groups. Finally, several biologically relevant metabolite ratios turned out to be significantly different between groups.

### 2. Characteristic Changes in Water-soluble Metabolites

#### 2.1. Similar effects of CFA and CFA/SC-H injections on water-soluble metabolites

CFA and CFA/SC-H inoculations affected a number of water-soluble brain metabolites in similar ways. The concentrations of the following five metabolites were decreased in inoculated rats vs. controls: taurine (tau), choline (cho), succinate (suc), *N*-acetylaspartate (NAA) and *scyllo*-inositol (scy-Ins) (Fig. 4, Table 1). The cytoprotective antioxidant, tau, is a an aminosulfonic acid ubiquitous in mammalian tissues, and plays a role in osmoregulation, membrane stabilization and modulation of calcium signaling [28]. NAA is an osmolyte almost exclusively localized in neurons; low NAA levels are considered to be markers of neuronal suffering. NAA serves as a co-transporter of water molecules and constitutes the primary mechanism for the removal of metabolic water from myelinated neurons [29]. The substrate and degradation product of Cho-PLs, cho, is an essential nutrient and a precursor molecule for the neurotransmitter acetylcholine. Suc is a metabolite of the citric-acid cycle responsible for oxidative phosphorylation (OXPHOS), and scy-Ins is an osmolyte derived from glucose.

A case in point is tau whose concentrations were virtually identical in CFA and CFA/SC-H animals, and were significantly reduced in each of these groups vs. the Contr group. The tau decreases amounted to 22%, and were the most substantial effects found among the water-soluble metabolites quantified (significant at the P<0.05 level for both absolute and relative concentrations, Table S2). Concentration decreases in each inoculated group vs. the control group were also significant for cho and scy-Ins, whereas for suc and NAA differences reached significance for the CFA group only (Fig. 4, Tables 1 and S2).

In contrast to these metabolites, concentrations of the glycolytic end product, lactate (lac), were increased by 8% in the CFA and CFA/SC-H groups vs. the Contr group (Table 1) (significant for CFA; Fig. 4). Also β-hydroxybutyrate (BHB) was increased in inoculated animals vs. Contr (significant for CFA/SC-H; Table 1 and Fig. 4). BHB is a ketone body that constitutes an important fraction of fuel for consumption by the brain under the conditions of certain diets, fasting, or vigorous exercise [30], [31]. Differences in brain concentrations of all of these metabolites (tau, cho, suc, NAA, scy-Ins, lac and BHB) showed statistical significance for CFA and CFA/SC-H groups combined vs. Contr (Table S3 C and D).

#### 2.2. Reversal of CFA effects on water-soluble metabolites by co-injection with SC-H in emulsion

Several CFA/SC-H metabolite concentrations, notably suc and scy-Ins levels, laid between the corresponding Contr and CFA values (Fig. 4). For aspartate (asp), an amino acid that may in part stem from NAA degradation, and U2 (an unassigned metabolite), almost identical concentrations were measured in the brains of Contr and CFA/SC-H rats. These results show that co*-*injection of SC-H with CFA lead to a partial or complete inhibition of effects caused by CFA alone on asp, suc, scy-Ins and U2 levels. This inhibition is not necessarily based on enzyme inhibition. In this report, we use the term inhibition solely to express that certain metabolic effects caused by CFA are abrogated (complete inhibition) or attenuated (partial inhibition) when CFA is co-injected with SC-H. In the case of a minor unidentified metabolite, U1, CFA and CFA/SC-H even have opposite effects (not significant, Table 2 B).

**Table 2:** Pathways and potential biochemical/biological effects associated with brain metabolites of rats inoculated with CFA or CFA/SC-H.

Inoculation	metabolic changes	metabolic pathways	biological functions	potential biological effects of inoculation
CFA/SC-H or CFA	tau↓, NAA↓ myo-Ins↑	osmolyte uptake and release	osmoregulation of neurons (NAA, tau) and astrocytes (myo-Ins, tau)	astrocyte hypertrophy for CFA/SC-H
CFA/SC-H or CFA	PL_tot_↑, cho↓	PL synthesis and degradation	structure of membrane matrix, source of signaling molecules	membrane PL accumulation; loss of tissue water due to dehydration
CFA/SC-H or CFA	Etn-PL↑, C-/E-PL↑	Etn-PL vs. Cho-PL turnover	Etn-PL: source of pro-inflammatory agents	increased potential to produce inflammatory signaling molecules; altered PL remodeling related to BBB impairment?
CFA/SC-H or CFA	lac↑, suc↓	glycolysis, citric acid cycle	balance between fermentative glycolysis and OXPHOS	increased glycolysis, decreased OXPHOS, possibly due to hypoxia owing to impaired microcirculation
CFA/SC-H ((CFA))	BHB↑	ketone body metabolism	replacing glucose when fuel stores depleted	depletion of fuel stores, possibly due to impaired microcirculation
CFA	asp↑	amino acid metabolism	protein synthesis and degradation, NAA metabolism	increased NAA degradation (impaired neurons); increased BBB permeability?

Metabolite levels increased (decreased) in the brains of inoculated vs. control rats are indicated by an upward (downward) arrow. For abbreviations see text, and Tables S1 D and S4.

The relative similarity between the Contr and CFA/SC-H metabolite profiles is also reflected by the fact that pooling these two groups, and comparing the combined values for each metabolite with the corresponding CFA value (Table S3 A), yielded virtually as many significant differences as the comparisons presented above in Section Results 2.1, *i.e.* between pooled (CFA+CFA/SC-H) data on the one hand and Contr values on the other (Table S3 C). In addition, the ketogenic and glucogenic essential amino acid, isoleucine (iso-leu), exhibited the same trend to similar Contr and CFA/SC-H levels (borderline significance, Table S3 A and D).

By contrast, comparing pooled (Contr+CFA) data to CFA/SC-H results yielded significant differences only for BHB and the ratio between two phospholipid metabolites, phosphocholine/glycerophosphocholine (PC/GPC), besides U1 (Table S3 B). GPC is a phosphatidylcholine (PtdCho) degradation product whereas PC is both an anabolite and a catabolite of PtdCho (PtdCho function is described in the following section).

GPC, which is also an osmolyte, tended to be particularly low in CFA/SC-H rats (Tables S3 B and D, and S4 C), resulting in an increased PC/GPC ratio which has long been known as an indicator of a high potential for growth [27].

Overall, these findings indicate that with respect to water-soluble brain metabolite profiles the CFA/SC-H group is closer to the Contr group than is the CFA group. Further evidence that CFA/SC-H metabolite profiles are situated between Contr and CFA is provided by tests for linear trends. Most significant trends were found when groups were ranked as Contr → CFA/SC-H → CFA (Table S4 B). Unsurprisingly, ranking as Contr → CFA → CFA/SC-H yielded unique significance for one metabolite only (positive trend for BHB**)**, and borderline significance for two further metabolites, lac and valine (val), the latter being an essential branched amino acid similar to iso-leu. Further results for water-soluble metabolites are presented in Section S2.2.

Since tau, cho and scy-Ins brain concentrations were found to be dramatically reduced in animals injected with either CFA or CFA/SC-H when compared with controls, these metabolic changes are unspecific with respect to the underlying disease, i.e., AA with extra-articular inflammation or EAE. However, increased asp and U2 levels can be considered metabolic brain markers that specifically characterize AA and distinguish this condition from EAE and Contr (Fig. 4 and Tables 1, S2 A and B). Particularly increased BHB levels can be considered characteristic of EAE, although this brain metabolite is only moderately EAE-specific because BHB shows a clear trend toward increased levels also for animals with AA.

### 3. Characteristic Changes in Phospholipids

#### 3.1. PL accumulation following CFA and CFA/SC-H injections

The observed PL_tot_ increase in CFA and CFA/SC-H vs. Contr groups was primarily due to a rise in ethanolamine-containing PLs (Etn-PL); there was a significant linear trend towards gradually increasing absolute concentrations for the sum of all Etn-PLs when going from controls to CFA-injected rats, and on to CFA/SC-H rats. This trend was particularly strong for alkyl-acyl-phosphatidylethanolamine (AAPtdEtn) (Table S1 A). AAPtdEtn is an important source of arachidonic acid and other polyunsaturated fatty acids (PUFA). The group of Etn-PLs comprises some of the most abundant membrane PLs; the other large PL group is composed of the choline-containing PLs (Cho-PL). The sum of all Cho-PLs was less increased in inoculated rats (notably CFA) vs. controls than was the sum of Etn-PLs (Fig. 3).

Absolute Etn-PL concentrations were about 20% higher in CFA/SC-H than in Contr brains (significant, Fig. 3), while this difference was only about 10% based on relative Etn-PL concentration (not significant). Generally, for virtually all major PLs intergroup differences were less pronounced when expressed as relative concentrations as opposed to absolute concentrations. This finding is not surprising because cells can alter the PL profiles of their membranes only within narrow limits without compromising essential cell functions [32], [33], [34], and are equipped with efficient compensatory mechanisms counteracting enforced changes in PL head groups [35]. Consequently, absolute amounts of PLs per mg brain tissue were markedly more variable between groups than the relative abundance of specific PL classes (PL profiles). Specific perturbations of particular PL synthesis or degradation pathways that result solely in altered membrane PL composition would not lead to an overall PL increase, but only to changes in relative PL levels. Therefore, when specific changes in PL profiles caused by CFA or CFA/SC-H are to be analyzed, this is most adequately done by comparing relative rather than absolute PL levels. Accordingly, the following section refers exclusively to relative PL concentrations and PL ratios.

#### 3.2. Reversal of CFA effects on phospholipids by co-injection with SC-H in emulsion

In our linear-trend analyses, most significant results were found when groups were ranked as Contr → CFA/SC-H → CFA (Table S1 B). The PLs concerned were PtdCho and its derivatives, and the sum (PtdIns_sum_) of phosphatidylinositol (PtdIns) and a minor associated PL; this PtdIns-associated PL is most likely a PtdIns derivative. The relative concentrations of these PLs were decreased in CFA vs. Contr (Tables S5 A and B), and were increased in CFA/SC-H vs. CFA, resulting in CFA/SC-H levels that approach control levels (Figs. 3 and S3). These results show that co*-*injection of SC-H with CFA lead to a reduction (PtdCho) or complete abrogation (PtdIns_sum_) of effects that would be caused by CFA alone.

PtdCho is by far the most abundant Cho-PL, and is also the most abundant PL class in membranes. Its primary function in cells is to provide, together with other PLs, the membrane matrix, and thus to ensure the basic stability and function of cell membranes. Etn-PLs and PtdIns are both important sources of signaling molecules. In contrast to Cho-PL or Etn-PL *per se*, the ratio of Cho-PL over Etn-PL ( = C−/E-PL) was virtually identical in the CFA/SC-H and CFA groups, and was significantly decreased by ca. 12% vs. Contr (Fig. 3).

In summary, CFA/SC-H and CFA injections led to overall increased absolute PL levels, and these increases were particularly pronounced for Etn-PL values in the CFA/SC-H group. As a consequence, the C−/E-PL ratio was significantly decreased following CFA/SC-H and CFA injection. Further PL results are presented in Section S2.3.

### 4. Neurohistopathology and Immunohistochemistry Results

**Figure 5:**
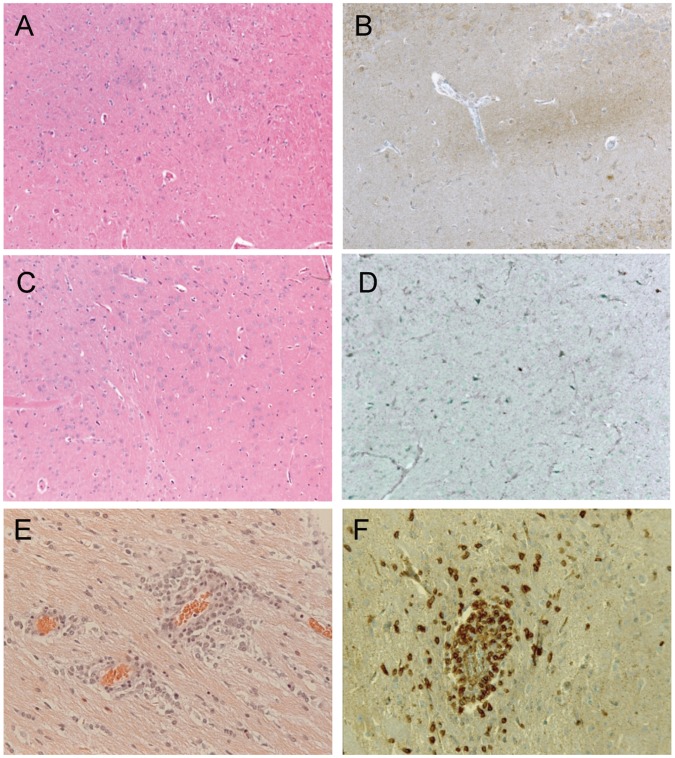
Perivascular cell infiltrates in the CNS of CFA/SC-H rats. Left column: hematoxylin-eosin staining. Right column: Immunoreactivity to CD3. No cell infiltration was detected in the brain sections of control and CFA rats (A, C and B, D respectively). Perivascular cell infiltrates were found in the midbrains of the CFA/SC-H rats (E). CD3 T lymphocytes were identified in cell infiltrates in the white matter (F).

Major perivascular cell infiltrates were found in the midbrain and hindbrain (Fig. 5 E and F), and at the 3 levels of the spinal cord (not shown) of the EAE-rats examined. In this group, CD3 T lymphocytes were identified in cell infiltrates, and were disseminated in the white matter of the hind brain, and, in particular, in the spinal cord. However, very little if any demyelination was observed in EAE rats. Cell infiltration and demyelination were absent from the CNS of CFA-injected and control animals (Fig. 5 A–D).

GFAP and IBA1 immunoreactivity in the cervical spinal cord and brain sections from rats in the control group identified astrocytes and microglia, respectively, and the morphology, location, and numbers of these positive cells were within normal limits in both Contr rats subjected to IHC (Fig. 5 A–D). Similarly, the morphology, location, and numbers of astrocytes and microglia in spinal cord and brain sections from rats of the CFA group were generally within normal limits. Compared to rats in the Contr group, there appeared to be minimal astrocytic hypertrophy in the white matter of the cervical spinal cord in two of the four CFA-inoculated rats examined by IHC. The apparent hypertrophy was due to slightly greater staining of the cell bodies and thickening of the cell processes. However, neither of the two brains from this group showed signs of hypertrophy of astrocytes or microglial cells. Consequently, the CFA group was characterized by borderline astrocytosis in the cervical spinal cord, and absence of microgliosis from cervical spinal cord and brain.

**Figure 6:**
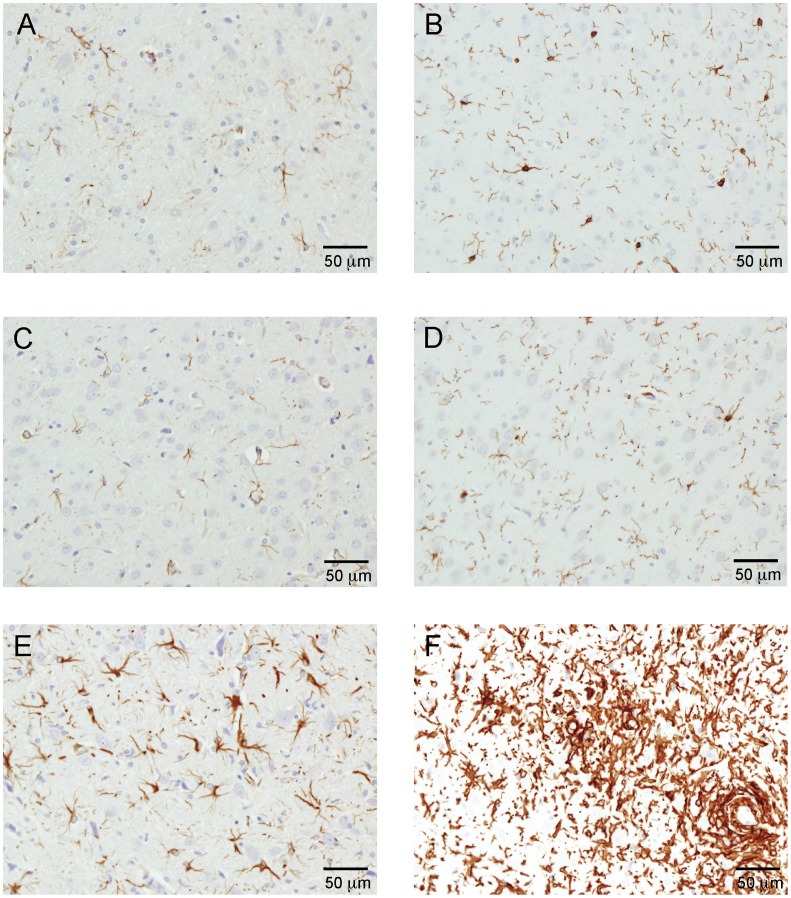
GFAP and IBA 1 immunoreactivity in brain sections. Images A, C and E illustrate GFAP immunoreactivity in brain sections from control, CFA and CFA/SC-H rats, respectively. Astrocytes appear similar for control and CFA rats (A and C, respectively). Reactive astrocytosis in the CFA/SC-H rat brain is depicted in image E. Images B, D and F illustrate IBA 1 immunoreactivity in brain sections from control, CFA and CFA/SC-H rats, respectively. Microglial cell size and number appear to be similar in the control and CFA rat brain (B and D, respectively). The reactive microgliosis in the CFA/SC-H rat brain is depicted in image F. Immunoreactivity for microglial cells is particularly dense in perivascular areas (lower right of image F).

In sharp contrast, astrocytes and microglia were clearly reactive in brain and cervical spinal cord sections from rats of the EAE group (Fig. 6 E–F). There was multifocal to diffuse hypertrophy of astrocytes in the gray and white matter of the spinal cords of all EAE animals examined by IHC. The reactive astrocytes were primarily located in the medulla and the area of the deep cerebellar nuclei. Increased numbers of hypertrophic microglia were present in all spinal cords and in the brain examined from EAE rats. The reactive microgliosis was multifocal, extensive, and marked in all of the spinal cords examined by IHC. Microgliosis was most prominent in the perivascular gray matter of the spinal cord. Astrocytes and microglia were hypertrophic, and microglial cells were increased in numbers. Consequently, astrocytes and microglia were reactive in the spinal cords and brains from rats of the EAE group.

## Discussion

This study presents, for the first time, a comprehensive brain metabolome analysis of comparing biochemical processes involved in EAE induced by CFA/SC-H injection, and in AA induced by CFA injection. The former is an inflammatory disease of the CNS; the latter notably includes articular autoimmune inflammation and major periarticular inflammation. AA is known to strongly impact on pain perception, and directly or indirectly may engage glial activation [16].

As was to be expected, the CNS of EAE rats was characterized by major infiltration of inflammatory cells, in part CD3+ T lymphocytes, and by marked microglia and astrocyte activation. In sharp contrast, these features were not detected in the CNS of rats with AA. Nonetheless, the brain metabolite profiles of the two injected groups (i) shared many characteristics that distinguished these groups from controls, and (ii) differed only with respect to a limited number of characteristics. The finding that many metabolite levels in both CFA and CFA/SC-H-inoculated groups were changed roughly by the same amount when compared with those of healthy controls would be consistent with either of two alternative hypotheses: (i) CFA is the component responsible for the most conspicuous metabolic changes in our EAE model, or (ii) CFA and CFA/SC-H injections provoke different physiological responses that converge toward common effects with respect to the metabolite levels in question. Several potential mechanisms are discussed in the subsequent sections; further interpretations are presented in Section S3. The most essential metabolic characteristics and their interpretations in terms of biological effects are compiled in Table 2.

### 1. Metabolism Involved in Osmoregulation is Equally Altered by Injection of CFA and CFA/SC-H

We found that two important organic osmolytes, taurine and, to a lesser extent, neuron-specific NAA were consistently reduced in the brains of rats injected with CFA or CFA/SC-H. Osmolyte loss from cells can provoke cell shrinkage. However, because IHC results did not reveal brain cell shrinkage in CFA or CFA/SC-H, we conclude that the osmolyte loss observed either was not sufficient to cause cell shrinkage, or was compensated for by an uptake of osmolytes not detectable by ^1^H NMR spectroscopy, primarily alkali ions.

The modest trend of the astrocytic marker, *myo*-inositol (myo-Ins), to increase after inoculation may indicate moderate growth of brain astrocytes, a notion supported by our IHC findings for the CFA/SC-H group, but not for the CFA group (Section S2.2). It was also reported that CFA-induced peripheral inflammation may raise mild to moderate microglial and astrocytic activation in the midbrain, the thalamus [11] and the anterior cingulated cortex [13], among other locations in the CNS [12]. However, in the CFA group no microglial activation was detected at the locations in which these features were detected in the EAE group, and minimal astrocytic activation was only present in the cervical cord but not in the brain. This suggests that either (i) cerebral effects caused by CFA injection did not affect the number and morphology of astrocytes and microglial cells, or that (ii) changes in these features did not exceed the detection threshold of the IHC method employed. We conclude that in the brain, the metabolic profile is more sensitive to CFA inoculation than the number and morphology of astrocytes and microglial cells, despite some minimal astrocytic effects found in the spinal cord.

### 2. Metabolism Involved in Phospholipid Production is Equally Altered by Injection of CFA and CFA/SC-H

CFA and CFA/SC-H injections caused a significant increase in total PL indicating a relatively increased presence of membranous structures in brain tissue. In addition, cho, a molecule involved in the metabolism of the PL PtdCho, was decreased in both inoculated groups vs. controls. The observed cho decrease may thus be due to increased cho consumption for membrane PL synthesis. Since Cho-PL and Etn-PL are the most abundant membrane PLs, relative variations in Cho-PL and Etn-PL levels reflect altered membrane PL composition due to CFA and/or SC-H effects on membrane PL synthesis and/or turnover.

Generally, increased PL levels may indicate (i) an increased amount of cells per mg brain tissue, resulting in hyperplasia; (ii) enlarged brain cells (hypertrophy); (iii) increased amounts of myelin, a particular type of membranous structure; or (iv) increased PL synthesis for incorporation into plasma and organelle membranes. In addition, increased PL_tot_ values (µmol/g tissue wet weight) may also be the result of (v) reduced tissue water content due to atrophy or dehydration. Atrophy is quite common in MS patients where it is the net result of irreversible and destructive pathological processes [36]. Loss of weight and dehydration are common features before and during clinical EAE. Painful stress is frequently associated with dehydration as animals are reluctant to drink [37], and pain due to the inflammation of the hind paw and the joints may have caused sufficient stress to reduce water intake. These factors may have caused brain dehydration in the CFA group, resulting in increased PL_tot_. The absence of concomitantly increased W_tot_ values might be explained by a release of water-soluble metabolites from brain tissue in the course of dehydration. Our histological results do not support the hypotheses of hyperplasia (i) and myelin increase (iii) discussed above, and significant hypertrophy (ii) has been observed only in the CFA/SC-H group, but not in the brains of the CFA group. In summary, both increased PL synthesis (iv) and dehydration (v) may be responsible for the observed PL_tot_ increase in inoculated animals, although the extent to which these processes contribute remains to be investigated. Further potential stress effects are discussed in Sections Discussion 3. and 4, whereas Section S3.2 describes additional hypothetical CFA and CFA/SC-H effects on PL metabolism.

### 3. Fermentative Glycolysis is Similarly Changed by Injection of CFA and CFA/SC-H

The global process of fermentative glycolysis, i.e. the conversion of glucose to lactate, is affected in similar ways by CFA and CFA/SC-H injections. This is indicated by virtually identical lactate levels in these two groups, which are increased vs. Contr values. As explained in Section S2.2.1, in our experiments the amount of lac measured reflects the entire pool of glycolytic substrates present in the brain *in vivo*. OXPHOS is the dominating mechanism of ATP production in brain, but under conditions of hypoxia, energy conversion has to rely on fermentative glycolysis, a much less efficient process that produces approximately 16 times less ATP for each glucose molecule than OXPHOS. Reduced OXPHOS is likely accompanied by a reduced bioenergetic status, in accordance with our previous findings for the brain of rats with adoptive EAE [3]. Reduced concentration of suc, a metabolite of the citric-acid cycle responsible for OXPHOS, in the CFA and CFA/SC-H vs. Contr groups also supports the hypothesis of reduced OXPHOS.

Hypoxia-like metabolic injury is a pathogenic component in a subset of inflammatory brain lesions [38]. Both vascular pathology and metabolic disturbances induced by toxins of activated macrophages and microglia are thought to be responsible for such lesions in MS [38]. Impaired microcirculation in MS has also been associated with lactate accumulation in cerebrospinal fluid [18]. Thus, the increased glycolytic and reduced OXPHOS activity suggested by increased lactate levels in rat brain tissue may be due to microvascular lesions following CFA and CFA/SC-H inoculations. A role for macrophages and activated microglia is unlikely in CFA rats because neither cellular infiltration and nor activation of microglia have been evidenced.

Moreover, stress due to the effects of CFA injection, described in Section Discussion 2., may also affect lac and BHB levels in rat brain. Extracellular lactate accumulation has been observed in the striatum and hippocampus of rats under conditions of emotional stress [39]. It has been hypothesized that stress induces glycogenolysis and lactate export from astroglial cells via neurotransmitter or hormonal related processes [40]. These mechanisms may contribute to the observed lactate increase in both CFA and CFA/SC-H-injected rats. As an alternative to glucose, ketone bodies such as BHB are important as fuel for consumption by the brain under conditions of depleting fuel stores.

### 4. AA-induced Stress and Alterations of the BBB may Account for Differential Effects of CFA and CFA/SC-H Injection on Glucose Derivatives and Amino Acids

While NAA, tau, lac and PLs effects caused by CFA and CFA/SC-H injection were very similar (Sections Discussion 1 to 3), other metabolic effects were more pronounced for CFA than they were for CFA/SC-H. Metabolic results were particularly conspicuous for (i) the glucose derivatives, suc and scy-Ins, and (ii) the amino acids, asp and ile. For each of the these metabolites, CFA/SC-H levels were closer to Contr values than were CFA levels, regardless of the direction of change, i.e. whether metabolite concentrations were increased or decreased in injected rats vs. controls. The same tendency was observed for another amino acid, glutamine (gln), showing slightly reduced levels vs. controls in the CFA group but not in the CFA/SC-H group (Fig. S4). At present, there is no straightforward explanation for these metabolic differences between the CFA and CFA/SCH groups, and only hypotheses may be proposed. Inflammatory pain, including pain caused by CFA-induced inflammation, has been reported to increase BBB permeability [41], [42]. Apart from genetic factors influencing the quantitative and qualitative aspects of immune responses, susceptibility to disease is modulated by the activity of the hypothalamic-pituitary-adrenal (HPA) axis and by microbial agents [43]. Although Lewis rats are known to display reduced response to stress compared with strains resistant or less sensitive to AA [44], even reduced response to stress, in particular caused by hyperalgesia, might be capable to result in adrenocorticotropic hormone and glucocorticoid production. Cortisol, in turn, is generally known to have various metabolic effects by increasing blood sugar through gluconeogenesis, and by aiding in fat, protein, and carbohydrate metabolism [45]. This mechanism may explain why in our study certain brain metabolic effects were more pronounced in AA than in EAE.

Whereas only occasional reports indicate that CFA can increase BBB permeability in the CNS, this phenomenon has been consistently observed in EAE. Markedly increased BBB permeability stands as a hallmark of EAE [3], [10], [41], [46], and should affect brain metabolite patterns. The BBB consists of tight junctions between endothelial cells, a thick basement membrane and astrocyte end-feet. These structures contain membranes based on a PL matrix with proteins inserted; PLs and proteins also determine the (supra)molecular structure of myelin. Therefore, not only any damage to the BBB but also its spontaneous repair may provoke alterations in PL and protein metabolism, as may demyelination and remyelination. Consequently, changes in amino acid concentrations may in principle reflect perturbations in protein synthesis and degradation linked to BBB and myelin alterations. However, taking into consideration that only slight demyelination has been detected for CFA/SC-H and none for CFA rats, it is unlikely that myelin perturbations contributed significantly to changes observed in amino acid and PL concentrations in the brain. By contrast, CFA and CFA/SC-H affect the BBB differentially, and these effects may be superimposed on the putative metabolic cortisol mechanisms.

### Conclusion

Using a metabolomic strategy, we have studied effects of two different inflammatory diseases on brain metabolism in a Lewis rat model: (i) EAE involving CNS inflammation, generated by CFA/SC-H injection, and (ii) AA with major extra-articular inflammation, generated by CFA injection only. Interestingly, both inflammatory diseases caused similar (though not identical) alterations in brain metabolic profiles. Several metabolic effects that were qualitatively similar for both inoculated groups, were more pronounced in rats with AA than in rats with CNS inflammation. Besides osmolytes and several glycolytic metabolites, Etn-PLs known as sources of inflammatory agents were increased in both inoculated groups. The similarity of CFA and CFA/SC-H effects on brain metabolism on the one hand, and the occurrence of cellular infiltration in the brains of CFA/SC-H-inoculated, but not of CFA-inoculated animals on the other suggest that the mechanisms responsible for the metabolic changes observed do not require the presence of inflammatory cells in the brain. Our findings open new avenues for future studies aimed at demonstrating whether brain metabolic effects provoked by AA are pain/stress-mediated and/or due to the presence of systemic proinflammatory molecules. Moreover, the possibility that CNS and AA may act through separate pathways that converge toward similar brain metabolic processes should be explored. Regardless of the nature of these mechanisms our results indicate that an inflammatory brain metabolite profile does not necessarily indicate the existence of cerebral inflammation; the presence of an extra-cerebral inflammation such as AA cannot be ruled out. This finding may be relevant for a broad variety of studies involving various types of inflammation in experimental animals. It may also be of interest for clinical studies of inflammatory diseases, e.g. by using metabolic *in-vivo* magnetic resonance spectroscopy (MRS).

## Supporting Information

Figure S1 Multivariate analyses of relative concentrations of PLs. Markers for results from individual animals are color-coded: black squares (control group), green filled circles (CFA-injected group), and red filled circles (CFA/SC-H-injected group). Representations of the first two (top left) or three (top right) principal components are shown as obtained from PCA analyses (unsupervised). The 3-D PCA plots also contain rays showing the directions of PL concentrations in the 3-D space (not annotated). The canonical plot (bottom) was obtained from LDA (supervised). Each multivariate mean is a labeled ellipse whose centroid is marked by a+sign. The size of each ellipse corresponds to a 95% confidence limit for the mean (CL ellipse). Distances between centroids are normalized to 1 for the shortest distance in each LDA plot.
(TIFF)

Figure S2 Multivariate analyses of relative concentrations of water-soluble metabolites. Markers for results from individual animals are color-coded as described in the legend to Fig. S1. Principal components and LDA are also presented as described for Fig. S1.
(TIFF)

Figure S3 Relative concentrations of several rat brain PLs in control animals, and in animals inoculated with CFA or CFA/SC-H (means and standard errors). Asterisks indicate statistically significant differences between the CFA and the control group, or between groups connected by lines. Although trends for differences in particular metabolite levels between groups can be clearly distinguished, many of these differences do not reach statistical significance. Therefore, statistical methods going beyond individual inter-group comparisons have been applied (Tables S1 to S6). PtdSer1 and PtdSer2 refer to the two partially overlapping signals making up the PtdSer resonance. The origin of this signal split is not currently known, but is probably due to the presence of fatty acid chains with different amounts and positions of double bonds. This split is more pronounced for PtdSer than it is for ethanolamine-containing PLs (Fig. 2).
(TIF)

Figure S4 Relative concentrations of several water-soluble rat brain metabolites in control animals, and in animals inoculated with CFA or CFA/SC-H (means and standard errors). For the statistical significance of differences between groups see comments in the legend to Fig. S3.
(TIFF)

Table S1 Significant linear trends for relative (rel.) and absolute (abs.) PL concentrations for different rankings of control, CFA and CFA/SC-H-treated rats. Upward (downward) arrows indicate a trend toward increased (decreased) concentrations.
(DOC)

Table S2 Significant differences in relative (rel.) and absolute (abs.) brain metabolite concentrations obtained from pairwise comparisons between rats treated with CFA or CFA/SC-H, and control animals.
(DOC)

Table S3 Significant differences in relative (rel.) and absolute (abs.) brain metabolite concentrations obtained from pooled comparisons between rats treated with CFA or CFA/SC-H, and control animals.
(DOC)

Table S4 Significant linear trends for relative (rel.) and absolute (abs.) water-soluble metabolite concentrations for different rankings of control, CFA and CFA/SC-H-treated rats. Upward (downward) arrows indicate a trend toward increased (decreased) concentrations.
(DOC)

Table S5 Significant differences in relative (rel.) and absolute (abs.) brain PL concentrations obtained from pairwise comparisons between rats treated with CFA or CFA/SC-H and control animals.
(DOC)

Table S6 Significant differences in relative (rel.) and absolute (abs.) brain PL concentrations obtained from pooled comparisons between rats treated with CFA or CFA/SC-H and control animals.
(DOC)

Text S1 Further details concerning Materials and Methods, Results, Discussion and References.
(DOC)
