# Supplementary figures and images for: Cerebral Biochemical Pathways in Experimental Autoimmune Encephalomyelitis and Adjuvant Arthritis: A Comparative Metabolomic Study
Source: PLoS One. 2013 Feb 14;8(2):e56101. doi: 10.1371/journal.pone.0056101 (PMC3573043; doi:10.1371/journal.pone.0056101)

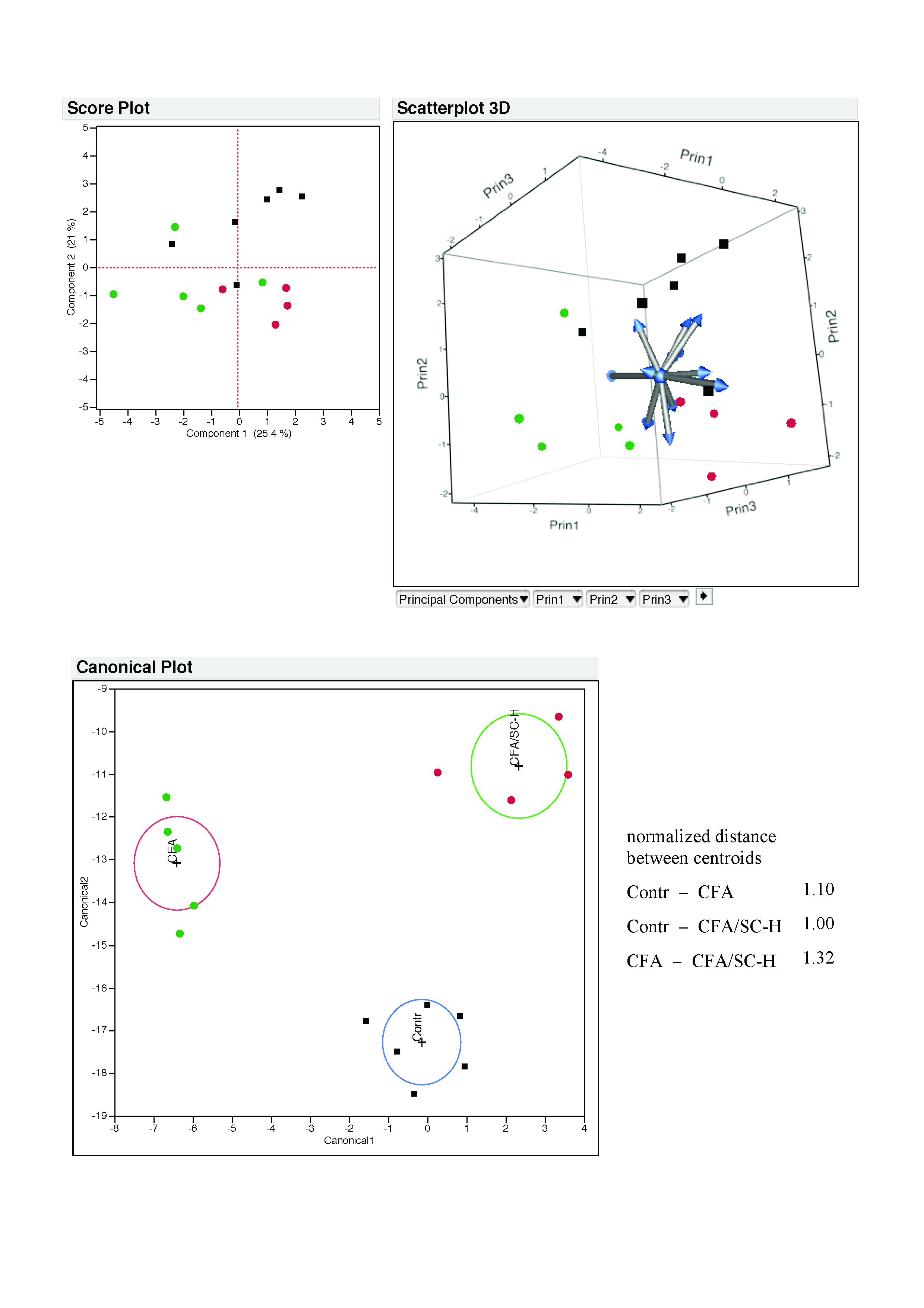

Supplement: Figure S1 — Multivariate analyses of relative concentrations of PLs. Markers for results from individual animals are color-coded: black squares (control group), green filled circles (CFA-injected group), and red filled circles (CFA/SC-H-injected group). Representations of the first two (top left) or three (top right) principal components are shown as obtained from PCA analyses (unsupervised). The 3-D PCA plots also contain rays showing the directions of PL concentrations in the 3-D space (not annotated). The canonical plot (bottom) was obtained from LDA (supervised). Each multivariate mean is a labeled ellipse whose centroid is marked by a+sign. The size of each ellipse corresponds to a 95% confidence limit for the mean (CL ellipse). Distances between centroids are normalized to 1 for the shortest distance in each LDA plot. (TIFF) [file pone.0056101.s001.tiff]

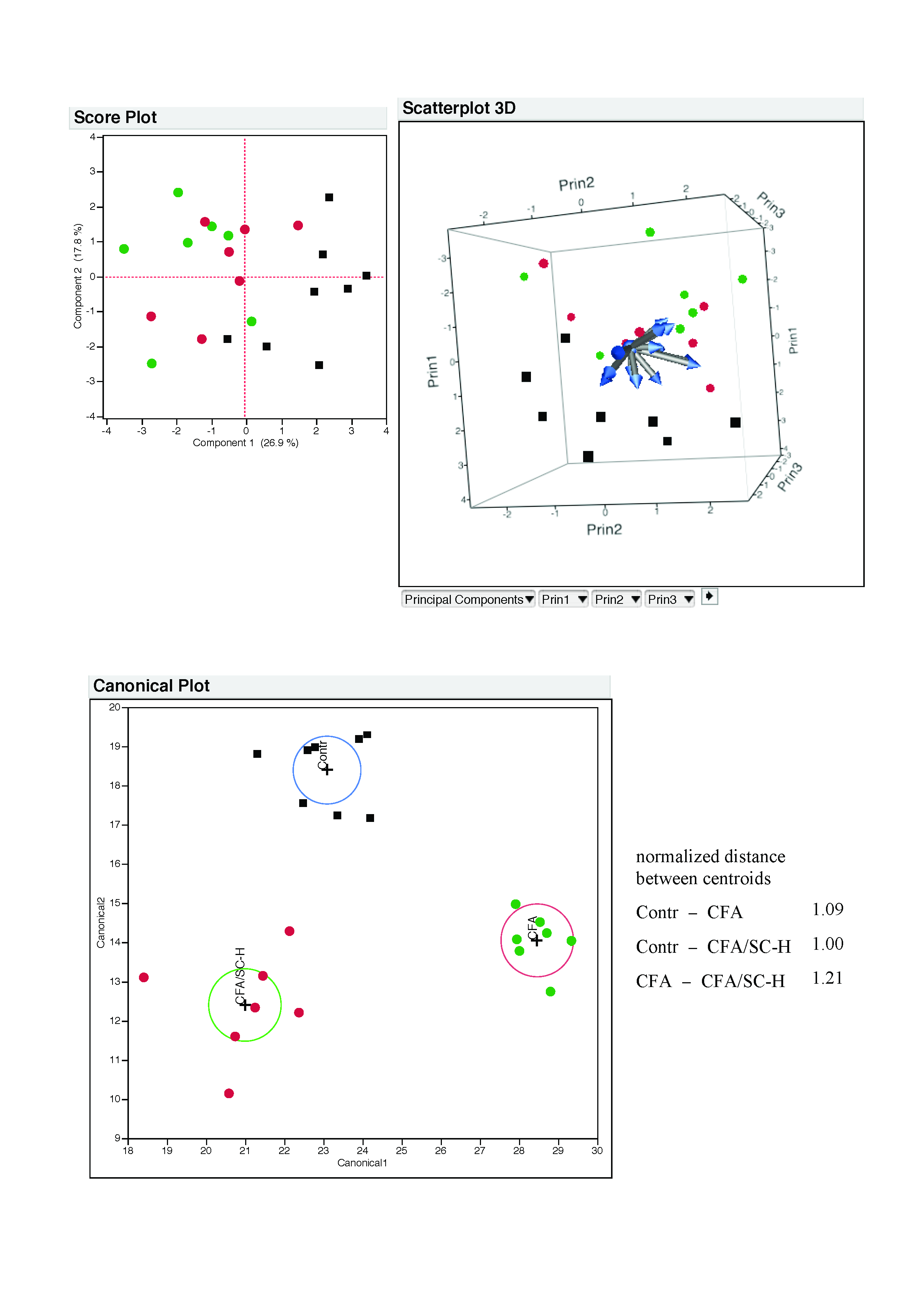

Supplement: Figure S2 — Multivariate analyses of relative concentrations of water-soluble metabolites. Markers for results from individual animals are color-coded as described in the legend to Fig. S1. Principal components and LDA are also presented as described for Fig. S1. (TIFF) [file pone.0056101.s002.tiff]

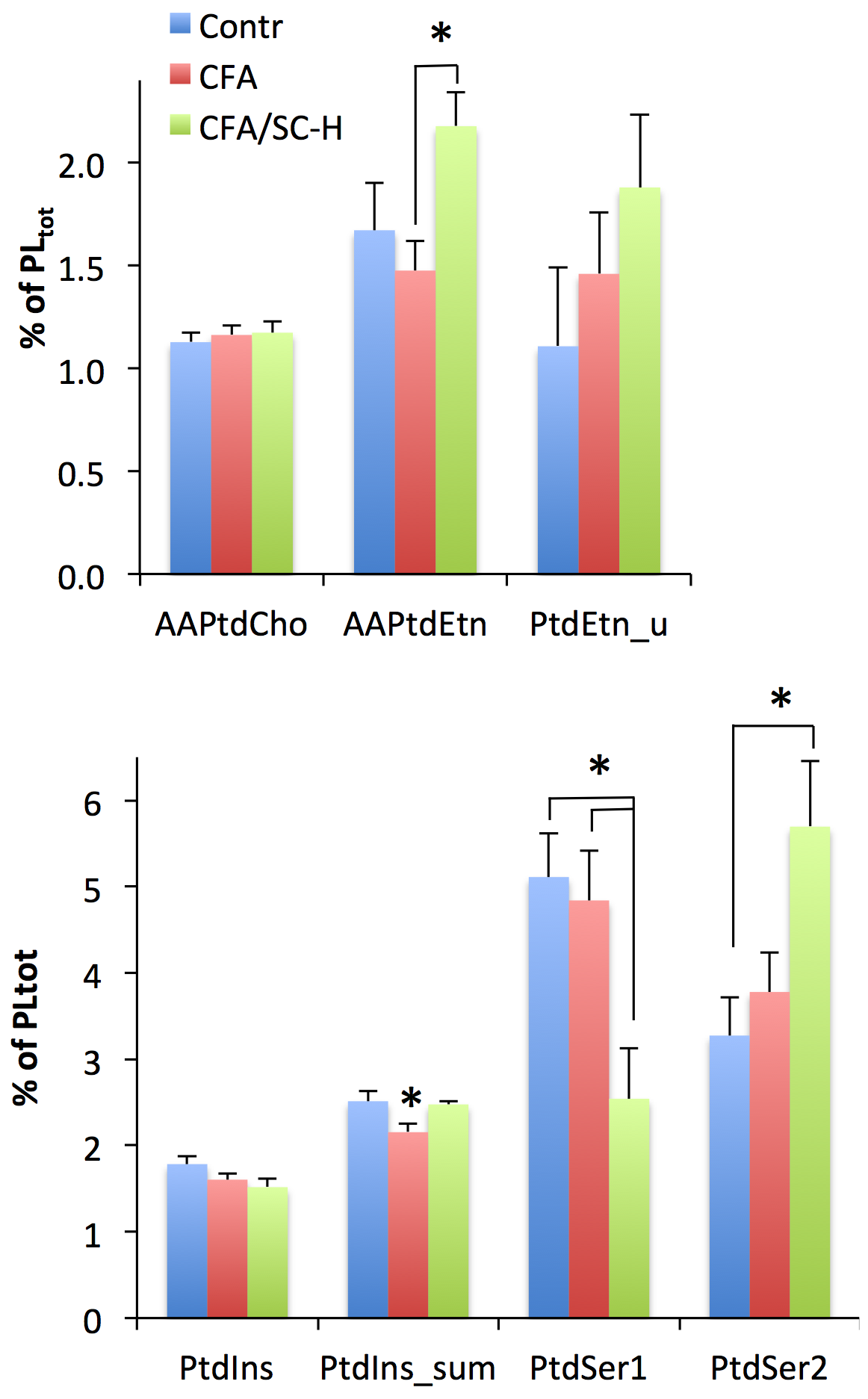

Supplement: Figure S3 — Relative concentrations of several rat brain PLs in control animals, and in animals inoculated with CFA or CFA/SC-H (means and standard errors). Asterisks indicate statistically significant differences between the CFA and the control group, or between groups connected by lines. Although trends for differences in particular metabolite levels between groups can be clearly distinguished, many of these differences do not reach statistical significance. Therefore, statistical methods going beyond individual inter-group comparisons have been applied (Tables S1 to S6). PtdSer1 and PtdSer2 refer to the two partially overlapping signals making up the PtdSer resonance. The origin of this signal split is not currently known, but is probably due to the presence of fatty acid chains with different amounts and positions of double bonds. This split is more pronounced for PtdSer than it is for ethanolamine-containing PLs (Fig. 2). (TIF) [file pone.0056101.s003.tif]

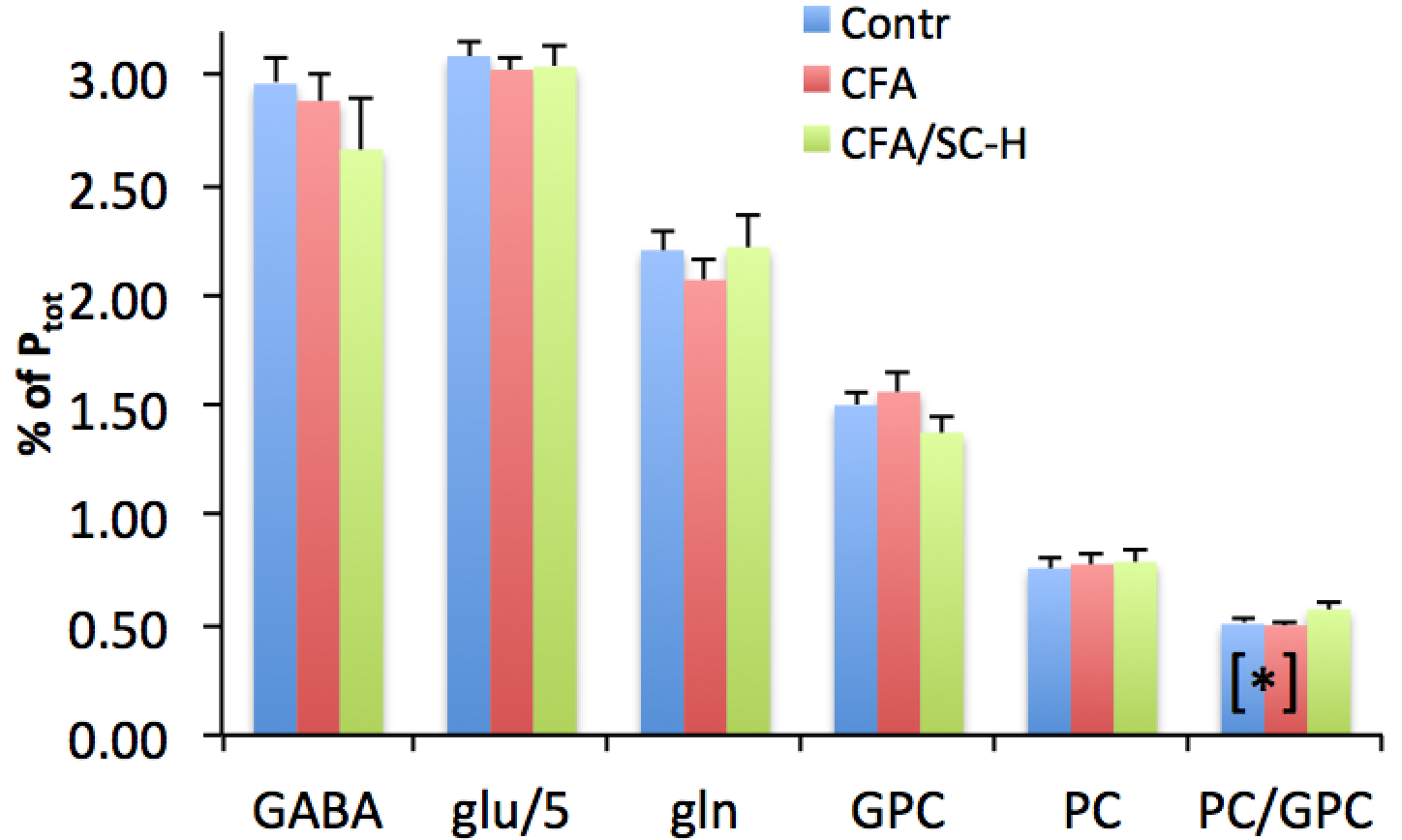

Supplement: Figure S4 — Relative concentrations of several water-soluble rat brain metabolites in control animals, and in animals inoculated with CFA or CFA/SC-H (means and standard errors). For the statistical significance of differences between groups see comments in the legend to Fig. S3. (TIFF) [file pone.0056101.s004.tiff]
