# Supplementary material for: Cerebral Biochemical Pathways in Experimental Autoimmune Encephalomyelitis and Adjuvant Arthritis: A Comparative Metabolomic Study
Source: PLoS One. 2013 Feb 14;8(2):e56101. doi: 10.1371/journal.pone.0056101 (PMC3573043; doi:10.1371/journal.pone.0056101)
Supplement: Table S1 — Significant linear trends for relative (rel.) and absolute (abs.) PL concentrations for different rankings of control, CFA and CFA/SC-H-treated rats. Upward (downward) arrows indicate a trend toward increased (decreased) concentrations. (DOC) [file pone.0056101.s005.doc]

S1 A) ranking: Contr → CFA → CFA/SC-H

|  | rel. | abs. |
| --- | --- | --- |
|  | | |
| *significant (******** *P < 0.05;* ******** *P < 0.01)* | | |
| AAPtdEtn |  | *******↑ |
| CL |  | *******↑ |
| Etn-PL |  | *******↑ |
|  | | |
| *borderline significant (0.05 < P < 0.13)* | | |
| AAPtdEtn | 0.0909 ↑ |  |
| Cho-PL |  | 0.0569 ↑ |
| AAPtdCho |  | 0.1227 ↑ |
| GroPtdCho |  | 0.0564 ↑ |
| PtdIns_sum_ |  | 0.1120 ↑ |
| PtdEtn_plas_ |  | 0.0783 ↑ |
| Etn-PL | 0.0719 ↑ |  |
| PtdGro | 0.1186 ↓ |  |
| C-/E-PL | 0.0605 ↓ |  |
| PL_tot_ |  | 0.0723 ↑ |
|  |  |  |
|  |  |  |
|  |  |  |
|  |  |  |

S1 B) ranking: Contr → CFA/SC-H → CFA

|  | rel. | | abs. |
| --- | --- | --- | --- |
|  |  | |  |
| *significant (******** *P < 0.05)* | | | |
| PtdCho | | *******↓ |  |
| PtdIns_sum_ | | *******↓ |  |
| PtdSer | |  | *******↑ |
| GroPtdCho | | ********↓ |  |
| C-/E-PL | | *******↓ |  |
| Cho-PL | | *******↓ |  |
| PL_tot_ | |  | *******↑ |
|  | |  |  |
| *borderline significant (0.05 < P < 0.13)* | | | |
| AAPtdCho |  | | 0.1194 ↑ |
| PtdEtn |  | | 0.0724 ↑ |
| CL |  | | 0.0576 ↑ |
|  |  | |  |

S1 C) ranking: CFA/SC-H → Contr → CFA

|  | rel. | abs. |
| --- | --- | --- |
|  |  |  |
| *significant (******** *P < 0.05;* ******** *P < 0.01)* | | |
| PtdIns_sum_ |  | ******* ↓ |
| AAPtdEtn | ******* ↓ | ******* ↓ |
| GroPtdCho |  | ******* ↓ |
|  |  |  |
| *borderline significant (0.05 < P < 0.13)* | | |
| PtdIns_sum_ | 0.0507 ↓ |  |
| PtdGro | 0.0916↑ | 0.0577↑ |
| GroPtdCho | 0.0587 ↓ |  |

| S1 D) Phospholipids quantitated in rat brain | |
| --- | --- |
| *choline PLs* | |
| 1) PtdCho | phosphatidylcholine |
| 2) AAPtdCho | alkyl-acyl-phosphatidylcholine |
| 3) GroPtdCho | sum of (1) + (2) + minor PtdCho derivatives such as plasmalogen |
| 4) Cho-PL | sum of choline PLs = (3) + sphingomyelin (SM) |
| *ethanolamine PLs* | |
| 5) PtdEtn | phosphatidylethanolamine |
| 6) AAPtdEtn | alkyl-acyl-phosphatidylethanolamine |
| 7) PtdEtn_plas_ | phosphatidylethanolamine plasmalogen |
| 8) PtdEtn_u | unassigned PL associated with PtdEtn |
| 9) Etn-PL | sum of ethanolamine PLs = (5) + (6) + (7) + (8) |
| C-/E-PL | ratio of (4) over (9) |
|  |  |
| *other PLs* |  |
| 10) CL | cardiolipin |
| 11) PtdIns | phosphatidylinositol |
| 12) PtdIns_sum_ | sum of 10 and associated PL |
| 13) PtdGro | phosphatidylglycerol |
| 14) PtdSer | phosphatidylserine |
| 15) PtdA  16) PtdInsP  17) PtdInsP_2_ | phosphatidic acid  phosphatidylinositol monophosphate (PIP)  phosphatidylinositol diphosphate (PIP_2_) |
| several unassigned PLs |  |
| PL_tot_ | sum of all PLs quantitated |
|  |  |
