# Supplementary material for: Cerebral Biochemical Pathways in Experimental Autoimmune Encephalomyelitis and Adjuvant Arthritis: A Comparative Metabolomic Study
Source: PLoS One. 2013 Feb 14;8(2):e56101. doi: 10.1371/journal.pone.0056101 (PMC3573043; doi:10.1371/journal.pone.0056101)
Supplement: Table S3 — Significant differences in relative (rel.) and absolute (abs.) brain metabolite concentrations obtained from pooled comparisons between rats treated with CFA or CFA/SC-H, and control animals. (DOC) [file pone.0056101.s007.doc]

S3 A) Comparison (Contr + CFA/SC-H) vs. CFA

|  | rel. | abs. |
| --- | --- | --- |
|  |  |  |
| *significant (P < 0.05)* | | |
| NAA | M-W ↑ |  |
| suc | t, M-W ↑ | t, M-W ↑ |
| scy-I | t, M-W ↑ | t, M-W ↑ |
| tau | t, M-W ↑ | t, M-W ↑ |
| U1 |  | t ↓ |
| U2 | t, M-W ↓ | t, M-W ↓ |
| asp | t, M-W ↓ | t, M-W ↓ |
|  |  |  |
| *borderline significant (0.05 < P < 0.13)* | | |
| lac | M-W: 0.1211 ↓ |  |
| NAA | t: 0.1038 ↑ | t: 0.1223, M-W: 0.0736 ↑ |
| iso-leu | t 0.0926 ↓ | t: 0.0655 ↓ |
| U1 | t: 0.0501, M-W: 0.0727 ↓ | M-W: 0.0824 ↓ |

S3 B) Comparison (CFA + Contr) vs. CFA/SC-H

|  | rel. | abs. |
| --- | --- | --- |
|  |  |  |
| *significant (P < 0.05)* | | |
| BHB | t, M-W ↓ | t ↓ |
| U1 | t, M-W ↑ | t, M-W ↑ |
| PC/GPC | t, M-W ↓ |  |
|  |  |  |
| *borderline significant (0.05 < P < 0.13)* | | |
| BHB |  | M-W: 0.0565 ↓ |
| GABA | M-W: 0.0574 ↑ | M-W: 0.1024 ↑ |
| asp | t: 0.1000, M-W: 0.0773 ↑ | t: 0.0680, M-W: 0.0773 ↑ |
| tau | t: 0.0980, M-W: 0.1024 ↑ | t: 0.0799, M-W: 0.0891 ↑ |
| cho | t: 0.0889 ↑ | t: 0.0973 ↑ |
| GPC | t: 0.1145, M-W: 0.1172 ↑ | t: 0.1296, M-W: 0.0891 ↑ |

S3 C) Comparison (CFA + CFA/SC-H) vs. Contr

|  | rel. | abs. |
| --- | --- | --- |
|  |  |  |
| *significant (P < 0.05)* | | |
| lac | t, M-W ↑ |  |
| NAA | t, M-W ↓ | t, M-W ↓ |
| suc | t, M-W ↓ | t, M-W ↓ |
| scy-Ins | t, M-W ↓ | t, M-W ↓ |
| cho | t, M-W ↓ | t, M-W ↓ |
| tau | t, M-W ↓ | t, M-W ↓ |
| BHB | t ↑ |  |
|  |  |  |
| *borderline significant (0.05 < P < 0.13)* | | |
| lac |  | t: 0.0746, M-W: 0.0564 ↑ |
| BHB | M-W: 0.0605 ↑ |  |
| U2 | t: 0.0591, M-W: 0.0699 ↑ | t: 0.0672, M-W: 0.0699 ↑ |
| [myo-Ins | t: 0.20 ↑] |  |

Upward (downward) arrows indicate increased (decreased) concentrations for the first vs. the second group compared in each column. All differences for which no P value is given were statistically significant at the P < 0.05 level in t tests and/or Mann-Whitney *U* tests as indicated (t and M-W, respectively). P values are given for differences that were borderline-significant. For abbreviations see text and Table S4 D.

S3 D) Overview

| (Contr + CFA/SC-H) vs. CFA | | (CFA + Contr) vs. CFA/SC-H | | (CFA + CFA/SC-H) vs. Contr | |
| --- | --- | --- | --- | --- | --- |
| rel. | abs. | rel. | abs. | rel. | abs. |
| NAA ↑  suc ↑  scy-Ins ↑  tau ↑  U2 ↓  asp ↓  *lac* ↓  *iso-leu* ↓  *U1* ↓ | suc ↑  scy-Ins ↑  tau ↑  U1 ↓  U2 ↓  asp ↓  *NAA* ↑  *iso-leu* ↓ | BHB ↓  U1 ↑  PC/GPC ↓  *GABA* ↑  *asp* ↑  *tau* ↑  *cho* ↑  *GPC* ↑ | BHB ↓  U1 ↑  *GABA* ↑  *asp* ↑  *tau* ↑  *cho* ↑  *GPC* ↑ | lac ↑  NAA ↓  suc ↓  scy-Ins ↓  cho ↓  tau ↓  BHB ↑  *U2* ↑  [myo-Ins ↑] | NAA ↓  suc ↓  scy-Ins ↓  cho ↓  tau ↓  *lac* ↑  *U2* ↑ |
| P < 0.05; *italics*: 0.05 < P < 0.13 | | P < 0.05; *italics*: 0.05 < P < 0.13 | | P < 0.05; *italics*: 0.05 < P < 0.07 | |
