# Supplementary material for: Cerebral Biochemical Pathways in Experimental Autoimmune Encephalomyelitis and Adjuvant Arthritis: A Comparative Metabolomic Study
Source: PLoS One. 2013 Feb 14;8(2):e56101. doi: 10.1371/journal.pone.0056101 (PMC3573043; doi:10.1371/journal.pone.0056101)
Supplement: Table S4 — Significant linear trends for relative (rel.) and absolute (abs.) water-soluble metabolite concentrations for different rankings of control, CFA and CFA/SC-H-treated rats. Upward (downward) arrows indicate a trend toward increased (decreased) concentrations. (DOC) [file pone.0056101.s008.doc]

S4 A) ranking: Contr → CFA → CFA/SC-H

|  | rel. | abs. |  |
| --- | --- | --- | --- |
|  |  |  |  |
| *significant (******** *P < 0.05;* ******** *P < 0.01;* ********* *P < 0.001)* | | | |
| scy-Ins | *******↓ | *******↓ |  |
| cho | *******↓ | *******↓ |  |
| tau | *********↓ | *********↓ |  |
| BHB | ********↑ | *******↑ |  |
|  |  |  |  |
| *borderline significant (0.05 < P < 0.13)* | | |  |
| lac | 0.0637 ↑ |  |  |
| val |  | 0.1256 ↓ |  |
| [myo-Ins | 0.18 ↑] |  |  |
|  |  |  |  |

S4 B) ranking: Contr → CFA/SC-H → CFA

|  | rel. | abs. |  |
| --- | --- | --- | --- |
|  |  |  |  |
| *significant (******** *P < 0.05;* ******** *P < 0.01;* ********* *P < 0.001)* | | | |
| lac | *******↑ |  |  |
| NAA | *******↓ | *******↓ |  |
| suc | *******↓ | *******↓ |  |
| scy-Ins | *********↓ | *********↓ |  |
| cho | *******↓ |  |  |
| tau | *********↓ | *********↓ |  |
| U2 | ********↑ | ********↑ |  |
| asp | *******↑ |  |  |
|  |  |  |  |
| *borderline significant (0.05 < P < 0.13)* | | | |
| lac |  | 0.1037 ↑ |  |
| cho |  | 0.0517 ↓ |  |
| iso-leu | 0.0863 ↑ | 0.1094 ↑ |  |
| asp |  | 0.0602 ↑ |  |
|  |  |  |  |

S4 C) ranking: CFA/SC-H → Contr → CFA

|  | rel. | abs. |
| --- | --- | --- |
|  |  |  |
| *significant (******** *P < 0.05)* | | |
| asp | *******↑ | *******↑ |
| scy-Ins | *******↓ |  |
| U1 | *******↑ | *******↑ |
| U2 | *******↑ | *******↑ |
| PC/GPC | *******↓ |  |
|  |  |  |
| *borderline significant (0.05 < P < 0.13)* | | |
| scy-Ins |  | 0.0730↓ |
| GPC | 0.0970↑ |  |
| BHB | 0.1074↓ | 0.0843↓ |
|  |  |  |

| S4 D) Water-soluble metabolites quantitated in rat brain | |  |
| --- | --- | --- |
| *osmolytes* | |  |
| 1) NAA | *N*-acetylaspartate |  |
| 2) myo-Ins | *myo*-inositol |  |
| 3) tau | taurine |  |
| 4) GPC | glycerophosphocholine |  |
| *amino and other acids* | |  |
| 5) lac | lactate (incl. a very small amount of threonine) |  |
| 6) BHB | β-hydroxybutyrate |  |
| 7) val | valine |  |
| 8) asp  9) leu | aspartate  leucine |  |
| 10) iso-leu | isoleucine |  |
| 11) suc | succinate |  |
| 12) ala | alanine |  |
| 13) ac | acetate |  |
| 14) NANA | *N*-acetylneuraminate |  |
| 15) gln | glutamine |  |
| 16) glu | glutamate |  |
| 17) gly | glycine |  |
| 18) GABA | γ-aminobutyrate |  |
|  |  |  |
| *phospholipid metabolites and other metabolites* | |  |
| 19) cho | choline |  |
| 20) PC | phosphocholine |  |
| 21) GPC | glycerophosphocholine (see also 4) |  |
| 22) scy-Ins | *scyllo*-inositol |  |
| 23) crn | creatine |  |
| PC/GPC | ratio of (20) over (21) |  |
| several unassigned metabolites such as U1 and U2 | |  |
|  | |  |
| W_tot_ | sum of all water-soluble metabolites quantitated |  |
|  |  |  |
|  |  |  |
