# Supplementary material for: Cerebral Biochemical Pathways in Experimental Autoimmune Encephalomyelitis and Adjuvant Arthritis: A Comparative Metabolomic Study
Source: PLoS One. 2013 Feb 14;8(2):e56101. doi: 10.1371/journal.pone.0056101 (PMC3573043; doi:10.1371/journal.pone.0056101)
Supplement: Table S5 — Significant differences in relative (rel.) and absolute (abs.) brain PL concentrations obtained from pairwise comparisons between rats treated with CFA or CFA/SC-H and control animals. (DOC) [file pone.0056101.s009.doc]

S5 A) Presentation for individual PLs

|  | rel. | abs. |
| --- | --- | --- |
| PtdCho | CFA < Contr (Tukey, Dunnett) |  |
| PtdIns_sum_ | CFA < Contr (Tukey, Dunnett) | CFA/SC-H > CFA (Tukey) |
| GroPtdCho | CFA < Contr (Tukey, Dunn, Dunnett) |  |
| Cho-PL | CFA < Contr (Tukey, Dunnett, Dunn) |  |
| C-/E-PL | CFA < Contr (Bonf.) |  |
| Etn-PL |  | CFA/SC-H > Contr (Bonf.) |
| AAPtdEtn | CFA/SC-H > CFA (Bonf.) | CFA/SC-H > Contr (Bonf.), and  CFA/SC-H > CFA (Bonf.) |
| PL_tot_ |  | CFA > Contr (Bonf.) |

All differences given in this table were statistically significant at the p < 0.05 level in the tests given in parentheses, except for Bonferroni tests (p < 0.1, underlined). For abbreviations see text and Table S1 D.

S5 B) Presentation for inter-group comparisons

| CFA vs. Contr | | CFA/SC-H vs. Contr | | CFA/SC-H vs. CFA | |
| --- | --- | --- | --- | --- | --- |
| rel. | abs. | rel. | abs. | rel. | abs. |
| PtdCho ↓  PtdIns_sum_↓  GroPtdCho ↓  Cho-PL ↓  C-/E-PL ↓ | PL_tot_ ↑ |  | Etn-PL ↑  AAPtdEtn ↑ | AAPtdEtn ↑ | PtdIns_sum_↑  AAPtdEtn ↑ |

Upward (downward) arrows indicate increased (decreased) concentrations for the first vs. the second group compared in each column (for relative and absolute concentrations). All differences given in this table were statistically significant at the p < 0.05 level in the tests given in parentheses, except for Bonferroni tests (p < 0.1, underlined).
