# Supplementary material for: Cerebral Biochemical Pathways in Experimental Autoimmune Encephalomyelitis and Adjuvant Arthritis: A Comparative Metabolomic Study
Source: PLoS One. 2013 Feb 14;8(2):e56101. doi: 10.1371/journal.pone.0056101 (PMC3573043; doi:10.1371/journal.pone.0056101)
Supplement: Table S6 — Significant differences in relative (rel.) and absolute (abs.) brain PL concentrations obtained from pooled comparisons between rats treated with CFA or CFA/SC-H and control animals. (DOC) [file pone.0056101.s010.doc]

S6 A) Comparison (Contr + CFA/SC-H) vs. CFA

|  | rel. | abs. |
| --- | --- | --- |
|  |  |  |
| *significant (P < 0.05)* | | |
| PtdCho | t, M-W ↑ |  |
| PtdIns_sum_ | t, M-W ↑ | t ↑ |
| GroPtdCho | t, M-W ↑ |  |
| Cho-PL | t, M-W ↑ |  |
|  |  |  |
| *borderline significant (0.05 < P < 0.13)* | | |
| PtdIns_sum_ |  | M-W: 0.0759 ↑ |
| PtdEtn |  | M-W: 0.0998 ↓ |
| AAPtdEtn | t: 0.1243, M-W: 0.1138 |  |
| PtdSer |  | t: 0.0853, M-W: 0.0658 ↓ |
| PtdGro |  | t: 0.0963 ↓ |
| PL_tot_ |  | t: 0.1299 ↓ |

S6 B) Comparison (CFA + Contr) vs. CFA/SC-H

|  | rel. | abs. |
| --- | --- | --- |
|  |  |  |
| *significant (P < 0.05)* | | |
| PtdIns_sum_ |  | t, M-W ↓ |
| AAPtdEtn | t, M-W ↓ | t, M-W ↓ |
| GroPtdCho |  | t ↓ |
|  |  |  |
| *borderline significant (0.05 < P < 0.13)* | | |
| CL |  | M-W: 0.1152 ↓ |
| PtdGro | t: 0.0793, M-W: 0.0890 ↑ | t: 0.1160 ↑ |
| GroPtdCho |  | M-W: 0.0542 ↓ |
| Cho-PL |  | t: 0.0723, M-W: 0.0863 ↓ |
|  |  |  |

S6 C) Comparison (CFA + CFA/SC-H) vs. Contr

|  | rel. | | | | abs. |
| --- | --- | --- | --- | --- | --- |
|  |  | | | |  |
| *significant (P < 0.05)* | | | | | |
| PtdCho | | | t, M-W ↓ |  | |
| CL | | |  | t, M-W ↑ | |
| GroPtdCho | | | M-W ↓ |  | |
| PL_tot_ | | |  | t, M-W ↑ | |
| C-/E-PL | | | t ↓ |  | |
| Etn-PL | | |  | t ↑ | |
| Cho-PL | | | t, M-W ↓ |  | |
|  | | |  |  | |
| *borderline significant (0.05 < P < 0.13)* | | | | | |
| PtdEtn_plas_ | |  | | t: 0.0669, M-W: 0.0631 ↑ | |
| PtdEtn | |  | | t: 0.0773, M-W: 0.1082 ↑ | |
| Etn-PL | | t: 0.0883 ↑ | | M-W: 0.0832 ↑ | |
| PtdA | |  | | t: 0.0927, M-W: 0.1195 ↑ | |
| PtdSer | |  | | t: 0.0647, M-W: 0.0631 ↑ | |
| GroPtdCho | | t: 0.0514 ↓ | |  | |
| C-/E-PL | | M-W: 0.0631 ↓ | |  | |

Upward (downward) arrows indicate increased (decreased) relative concentrations for the first vs. the second group compared in each column All differences for which no P value is given were statistically significant at the P < 0.05 level in t tests and/or Mann-Whitney *U* tests as indicated (t and M-W, respectively). P values are given for differences that were borderline-significant. For abbreviations see text and Table S1 D.

| (Contr + CFA/SC-H) vs. CFA | | (CFA + Contr) vs. CFA/SC-H | | (CFA + CFA/SC-H) vs. Contr | |
| --- | --- | --- | --- | --- | --- |
| rel. | abs. | rel. | abs. | rel. | abs. |
| PtdCho ↑  PtdIns_sum_↑  GroPtdCho↑  Cho-PL↑  *AAPtdEtn*↑ | PtdIns_sum_↑  *PtdEtn*↓  *PtdSer*↓  *PtdGro*↓  *PL_tot_*↓ | AAPtdEtn↓  *PtdGro*↑ | PtdIns_sum_↓  AAPtdEtn↓  GroPtdCho↓  *CL*↓  *PtdGro*↑  *Cho-PL*↓ | PtdCho ↓  GroPtdCho↓  C-/E-PL↓  Cho-PL↓  *Etn-PL* ↑ | CL ↑  Etn-PL ↑  PL_tot_ ↑  *PtdEtn_plas_*↑  *PtdEtn* ↑  *PtdA* ↑  *PtdSer* ↑ |
| P < 0.05; *italics*: 0.05 < P < 0.13 | | P < 0.05; *italics*: 0.05 < P < 0.12 | | P < 0.05; *italics*: 0.05 < P < 0.12 | |

S6 D) Overview
