# Supplementary material for: Cerebral Biochemical Pathways in Experimental Autoimmune Encephalomyelitis and Adjuvant Arthritis: A Comparative Metabolomic Study
Source: PLoS One. 2013 Feb 14;8(2):e56101. doi: 10.1371/journal.pone.0056101 (PMC3573043; doi:10.1371/journal.pone.0056101)
Supplement: Text S1 — Further details concerning Materials and Methods, Results, Discussion and References. (DOC) [file pone.0056101.s011.doc]

**Supporting Information**

**S1. MATERIALS AND METHODS**

S1.1. Sample preparation for metabolomic NMR spectroscopy

Animals were anesthetized by isoflurane inhalation, and were sacrificed by cervical dislocation before decapitation with a pair of scissors. The brain was swiftly and entirely removed through an incision in the skull, freeze-clamped, weighed, and stored at -80°C until extraction with methanol/chloroform/water (4:4:4 mL). The frozen brain was ground into small pieces in a mortar under liquid nitrogen, and the brain pieces were thoroughly mixed by stirring. After nitrogen evaporation, 250 to 350 mg brain tissue was weighed, added to 4 mL ice-cold methanol and immediately homogenized using a Polytron tissue homogenizer (Kinematica, Kriens-Lucerne, Switzerland). The sample was incubated on ice for 15 min, then 4 mL of ice-cold CHCl_3_ and 4 mL 4°C water were added sequentially, under vigorous shaking after each addition. Samples were thoroughly vortexed and placed at -20°C overnight for phase separation. Subsequent centrifugation at 11,000 rpm (ca. 13,000 g at max. radius) in a Sorvall Evolution RC centrifuge (Thermo Electron Corporation, Waltham, MA, USA) for 40 min at 4°C completed the phase separation. Solvents were evaporated from the chloroform/methanol phase under a nitrogen stream, and methanol was removed from the water/methanol phase by bubbling nitrogen through the solution while samples were kept on ice. The aqueous solution was lyophilized overnight, and both lyophilizate and lipid samples were stored at -80°C. For NMR spectroscopy, lipids were dissolved in an appropriate amount (1100 to 1400 μL) of a ^2^H‑chloroform/methanol/water solution as described elsewhere , to yield solutions of similar phospholipid concentrations for all samples. The lyophilizate was redissolved in 700 μL D_2_O, and pH was adjusted to 7.0 ± 0.05. The sample was placed in a 5‑mm NMR tube (528-PP; Wilmad, Buena, NJ, USA) containing a stem coaxial insert (2 mm O.D.) filled with a D_2_O solution (pH 7.7) of 20 mM sodium 3-(trimethylsilyl)-2,2',3,3'-tetradeuteropropionate (TSP-d_4_) for ^1^H NMR, or with 20 mM methylenediphosphonate (MDP) for ^31^P NMR, for chemical-shift referencing and quantitation. Chemicals were purchased from Sigma-Aldrich, except for a number of phospholipids used for MRS signal assignment (Doosan Serdary Research, Toronto, ON, CA).

S1.2. Calculation of relative metabolite concentrations

In addition to absolute metabolite concentrations, ratios of metabolite levels were calculated, most importantly molar fractions of all measured metabolites in a given spectrum, i.e.

.

Multiplication of the molar fractions by a factor of 100 yielded relative concentrations given in percent of total metabolite concentration. This calculation was performed separately for water-soluble metabolites and phospholipids. Comparing relative metabolite concentrations between groups has the advantage of directly revealing redistributions within the tissue metabolite pool, irrespective of changes in the absolute size of the total pool. Thus, such inter-group comparisons directly indicate shifts in metabolic channeling. In addition, evaluating relative concentrations eliminates potential errors introduced by sample handling such as weighing or pipetting; any loss in biological material that might occur (after sample homogenization) would affect all metabolites of a given measurement sample to the same extent, and would, therefore, not change relative concentrations.

S1.3. Statistics

Parametric tests such as Student's t test and analysis of variance with parametric post-hoc analysis are appropriate where the conditions of equal variances and normal distribution are met. However, tests for normal distribution do not give meaningful results when applied to small sample groups , as is the case in this study. To minimize any bias, we therefore tested the outcome of both parametric and non-parametric methods. In virtually all cases, these methods yielded consistent results for statistical significance of differences in metabolite concentrations, regardless of whether individual groups were compared against each other, or whether pooled comparisons were performed. Minor discrepancies occurred only for a few low-abundance metabolites; some inter-group comparisons yielded statistical significance in parametric tests while non-parametric tests exhibited only borderline significance (0.05 < P < 0.1). This was due to the fact that non-parametric tests such as the Mann-Whitney *U* or Kruskal-Wallis test (that are intrinsically conservative) were not sufficiently powerful to unambiguously demonstrate significant differences. The customarily applied threshold value for statistical significance, P < 0.05, was used in our analyses. However, an exception was made for the particularly conservative Bonferroni test; the statistics software employed here offers the possibility to enhance the power of this test by setting the threshold to P = 0.10, thus avoiding a high number of false negatives. Significance obtained in this way is clearly identified in all tables presenting statistical results (Tables 1 and S1 to S6), as is significance based on the Newman-Keuls test. The latter test is rather powerful at the P < 0.05 level used, but at the cost of a somewhat increased risk of false positives (both tests from Prism 5.0, GraphPad, San Diego, CA, USA). Significance by the Bonferroni test (P < 0.10) or by the Newman-Keuls test (P < 0.05) was referred to as borderline significance, as was significance based on the other tests used for 0.05 < P < 0.10, or close to 0.1.

In the context of our study, a direct interpretation of pooled comparisons is meaningful where the means of the individual groups to be combined are similar, and are clearly different from the means of the groups they are to be compared with. Thus, when applied judiciously, pooled comparison can increase the power of a statistical test by increasing sample size, while pooling data from widely differing groups might have the effect of obscuring existing differences.

Our post test for a linear trend, although less widely used than other parametric and non-parametric tests, is well established . It is based on calculating linear regression (yielding an r^2^ value) on group mean vs. group number, the latter depending on the order in which groups have been arranged. The null hypothesis tested is that there is no linear trend between group number and group mean. Consequently, the P value calculated in this test is the probability of observing, as a consequence of random sampling, an r^2^ value that is as high or higher than the calculated r^2^ value, with P < 0.05 being the threshold for statistical significance.

**S2. RESULTS**

S2.1. General metabolic trends

Based on the two-dimensional PCA plot for PLs (Fig. S1, top left), weak separation of the data clouds can be distinguished for all three groups, indicating moderately characteristic differences between PL profiles. Contr tend to be prevalent in the upper right, CFA in the lower left, and CFA/SC‑H in the lower right quadrant of this plot. However, there is significant overlap between these data clouds, which are somewhat better separated in the three-dimensional PCA plot (Fig. S1, top right). Note that data points for some animals are missing from these PCA plots (as well as from the canonical plot discussed below), because one or several PLs were immeasurable in their brain samples. A tentative exclusion of these PLs from PCA analysis allowed us to inspect data points for all animals, but did not improve group separation (data not shown). The canonical plot (Fig. S1, bottom) demonstrates that the brain PL profiles of the three groups compared were sufficiently different to distinguish quite well Contr, CFA and CFA/SC‑H rats when using supervised LDA (no misclassifications). The distances between any two multivariate means (ellipse centroids) were similar. The largest distance was found between CFA and CFA/SC‑H (best distinction based on PL levels), and the smallest distance between Contr and CFA/SC‑H (relatively poor distinction).

Based on the two-dimensional PCA plot for water-soluble metabolites (Fig. S2, top left), weak separation of the data clouds can be distinguished, akin to the situation described above for PLs. Contr tend to be prevalent in the right half of this plot, CFA in the left half, and CFA/SC‑H in the center-left. However, there is significant overlap between the data clouds, notably between CFA and CFA/SC‑H. Data points are hardly better separated in the three-dimensional PCA plot (Fig. S2, top right). The canonical plot (Fig. S2, bottom) demonstrates that the profiles of the water-soluble metabolites permit a clear distinction between Contr, CFA and CFA/SC‑H rats when using LDA (no misclassifications). The distances between any two multivariate means were similar, in analogy to the results obtained for PLs (preceding paragraph). Again, the largest distance was found between CFA and CFA/SC‑H (best distinction), and the smallest distance between Contr and CFA/SC‑H (relatively poor distinction).

S2.2. Characteristic changes in water-soluble metabolites

S2.2.1. Similarities between metabolic effects of CFA and CFA/SC‑H

The differences in tau, cho, suc and lac concentrations between Contr rats on the one hand and CFA and CFA/SC‑H rats on the other (Section Results 2.1) were statistically significant as determined by both parametric and non-parametric tests, in particular when based on relative concentrations (Table S3 C); for BHB, the increase for the combined relative CFA and CFA/SC‑H vs. Contr values was significant by the parametric Student's t-test and borderline-significant by the non-parametric Mann-Whitney *U* test. Note that a substantial part of the ate pool measured in brain extracts originates from anaerobic glycolysis due to warm ischemia caused by sacrificing the animal. Although our protocol ensures that the time that elapses before tissue freezing is kept to a minimum, glucose and glycolytic intermediates are overwhelmingly converted to lactate within less than one minute . Therefore, the amount of lactate measured in brain tissue extracts reflects the entire pool of glycolytic metabolites present in the brain prior to death, in addition to the *in-vivo* lactate pool.

Other metabolites showed trends similar to those mentioned above, but differences did not turn out to be significant. For instance, the neurotransmitters, glutamate (glu) and γ-aminobutyrate (GABA), tended to be less concentrated in the CFA and CFA/SC‑H groups than in controls (Fig. S4), akin to the neuronal marker, NAA (Fig. 4). The only osmolyte measured that tended to be increased in CFA and CFA/SC‑H vs. Contr was the astrocytic marker, myo-Ins (Table S3 C and D), although these differences failed to reach statistical significance.

S2.2.2. SC‑H co-injection reverses specific metabolic effects of CFA alone

While there is clear evidence that SC‑H co-injection with CFA inhibited suc and scy-Ins concentration effects that would be caused by CFA alone (Section Results 2.2), a similar effect was observed for the amino acid, gln (smallest values observed for the CFA group), but differences were not pronounced enough for statistical significance (Fig. S4). In the brain, astrocyte-produced gln is linked to neuron-produced glu via the glutamate-glutamine cycle which maintains an adequate glu supply.

For tau, scy-Ins, suc, asp and an unassigned metabolite, "U2", both the t-test and the Mann-Whitney *U* test yielded statistical significance, while for NAA the Mann-Whitney *U* test resulted in significance and the t test in borderline-significance for differences between the pooled (Contr + CFA/SC‑H) and CFA concentrations (relative concentrations, Table S3 D). These results confirm the relative similarity between the Contr and CFA/SC‑H concentrations of these metabolites when compared with the corresponding CFA values.

As pointed out in Section Results 2.2, most significant linear trends were found when groups were ranked as Contr → CFA/SC‑H → CFA (Table S4 B). The metabolites concerned were lac, U2 and asp (positive trends), as well as NAA, suc, scy-Ins, cho, and tau (negative trends). Most of these trends were significant for both absolute and relative concentrations. When the groups were ranked as CFA/SC‑H → Contr → CFA (Table S4 C), a new positive linear trend was observed for U1 (unknown metabolite, significant for absolute and relative concentrations) and a negative trend for PC/GPC; both trends were not significant for any other ranking.

In addition**,** *myo*-inositol (myo-Ins) was slightly increased in the CFA and CFA/SC‑H groups (Tables S4 A, S3 C and D), but owing to major standard errors this rise was not statistically significant and should be characterized as a rather weak trend (Tables S4 A and S6 C,D). Myo-Ins, a glucose-derived polyol forming the structural basis for a number of secondary messengers, is predominantly localized in astrocytes (astrocyte marker).

S2.3. Characteristic changes in phospholipids

S2.3.1. Overall PL behavior following CFA or CFA/SC‑H injection

The PL_tot_ increase observed as a consequence CFA or CFA/SC‑H injection was most significant when data from both injected groups were pooled and compared with the control group (Tables S6 C and D) or when linear trends were evaluated (Table S1 A and B), but borderline significance could also be demonstrated for CFA vs. controls (Tables S5 A and B).

Since absolute Etn-PL levels increased more than absolute Cho-PL levels, C-/E-PL ratios were reduced (-12 %) in inoculated animals when compared with controls (Fig. 3). As for PL_tot_, C-/E-PL differences were most significant (i) for linear trends (Tables S1 A and B), (ii) when CFA and CFA/SC‑H data were pooled (Tables S6 C and D), and (iii) for CFA vs. controls (Tables S5 A and B).

S2.3.2. Reversal of CFA effects on PLs by CFA/SC‑H co-injection

The similarity of certain Contr and CFA/SC‑H PL levels is reflected by the fact that pooling the PLs from these two groups, and comparing the combined values with the corresponding CFA values, yielded almost as many significant differences as comparisons between pooled (CFA/SC‑H + CFA) and Contr values (Table S6 D). Besides Cho-PLs and PtdIns_sum_, this applies to the Etn-PL, alkyl-acyl-PtdEtn (AAPtdEtn), albeit at borderline significance. CFA/SC‑H co-injection increased AAPtdEtn levels even far beyond Contr levels (Fig. S3), which is also reflected by significant linear trends for this PL when groups were ranked as CFA/SC‑H → Contr → CFA (Table S1 C). Also in comparisons between individual groups, AAPtdEtn was found to be more concentrated in CFA/SC‑H than in CFA rats (borderline significance, Fig. S3). Phosphatidylserine (PtdSer) is a particular case in that its relative concentration was constant across all three groups, although the contributions to PtdSer of two PtdSer sub-classes that were distinguishable in ^31^P NMR spectra, differed between the CFA/SC‑H group on the one hand and the Contr or CFA groups on the other (Fig. S3). Both Etn-PLs and PtdSer are rich in PUFAs and are located in the inner layer (the cytosolic side) of cell membranes, while PtdCho is rich in saturated and mono-unsaturated fatty acids and is located in the outer layer.

**S3. DISCUSSION**

S3.1. Potential cellular effects of CFA and CFA/SC‑H injection underlying alterations in the concentrations of the osmolytes, myo-Ins and NAA

Our metabolomic evidence of moderately increased astrocyte growth in CFA/SC‑H-rats, in conjunction with IHC results showing that astrocytes were hypertrophic, are in agreement with the fact that astrocyte swelling and proliferation are characteristic responses to inflammation observed during EAE . It has been reported that CFA-induced peripheral inflammation may cause mild to moderate microglial and astrocytic activation in particular in the L5 lumbar spinal cord (which presents the most intense activity), in addition to the midbrain, thalamus and anterior cingulated cortex (see Section Discussion 1). Lumbar spinal cords were not examined in CFA rats; therefore we cannot rule out the possibility that microglial and astrocytic activation has occurred at that level. The absence of IHC-detectable activation in the brains of CFA-injected rats may indicate that the modest myo-Ins increase observed in these animals may reflect a form of metabolic activation that is either independent of IHC-detectable astrocyte activation, or that precedes  . In conclusion, the observed decrease in the neuronal marker, NAA, in conjunction with the mild myo-Ins increase and the PL metabolite findings presented in our work, would be in agreement with a process involving neuronal suffering accompanied by moderate metabolic astrocyte activation.

S3.2. Potential effects of CFA and CFA/SC‑H injection on PL remodeling and phospholipase activity

Etn-PLs**,** being increased in both CFA and CFA/SC‑H brains vs. controls, are rich in arachidonic acid and other PUFAs, which in turn are substrates for the production of proinflammatory agents. Thus, the increased Etn-PL levels detected suggest an increased capacity of the brain to produce inflammatory signaling molecules regardless of whether the animals suffer from AA with significant peri-articular inflammation, or from EAE.

Systemic Mt-containing CFA can promote CNS inflammation, a process that may trigger PL remodeling potentially resulting in increased Etn-PL production to further drive the inflammatory process through increased eicosanoid synthesis . The importance of PL remodeling in inflammation has long been recognized . In line with this notion, brain Etn-PL levels were increased in the CFA group vs. Contr of our study, and, to an even higher degree, in the CFA/SC‑H group. The fact that no cellular infiltration was detected in the brains of CFA-injected rats does not preclude the possibility that brain inflammation may be mediated by signaling molecules such as cytokines; there is evidence that peripheral inflammation is associated with an immunological activation in the CNS in both humans and mice, as shown for rheumatoid arthritis, possibly via prostaglandin-mediated signaling across the intact BBB .

In neuroinflammation, phopholipases A_2_ and cyclooxygenases are stimulated, resulting in membrane PL breakdown and liberating arachidonic acid predominantly from Etn-PL. Phospholipase A_1_ activity is increased in the acute stage of EAE . Conversely, cytosolic phospholipase A_2_-deficient mice are resistant to EAE and to collagen-induced arthritis . Protecting cells from phospholipase A_2_ action has been suggested as anti-inflammatory treatment . Many mediators of inflammation are indeed derived from phospholipase-catalyzed PL degradation . These include a number of compounds synthesized from arachidonic acid and diacylglycerol (DAG), as well as platelet activating factor (PAF) synthesized from a 1-alkyl-2-lysophosphatidylcholine . In fact, PAF is a bioactive lipid acting on neuronal growth, glutamate exocytosis and other processes. Phospholipase A_2_ activity is also modulated by inositol 1,4,5-triphosphate, a degradation product of the PL, phosphatidylinositol 4,5-biphosphate . Phospholipase activity may also be involved in processes leading to PL accumulation in vacuoles. To summarize*,* there is ample evidence that the perturbations of Etn-PL metabolism measured under conditions of CFA/SC‑H inoculation closely resemble those caused by CFA-induced inflammation, and that both are most probably directly related to the production of inflammatory agents.

**References**

1. Lutz NW, Cozzone PJ (2010) Multiparametric optimization of (31)P NMR spectroscopic analysis of phospholipids in crude tissue extracts. 2. Line width and spectral resolution. Anal Chem 82: 5441-5446.

2. Lutz NW, Cozzone PJ (2010) Multiparametric optimization of (31)P NMR spectroscopic analysis of phospholipids in crude tissue extracts. 1. Chemical shift and signal separation. Anal Chem 82: 5433-5440.

3. Lilliefors HW (1967) The Kolmogorov-Smirnov test for normality with mean and variance unknown. J Amer Stat Assn 62: 399-402.

4. Sokal RR, Rohlf FJ (1995) Biometry. New York: Freeman.

5. Altman DG (1991) Practical Statistics for Medical Research. London: Chapman and Hall.

6. Ponten U, Ratcheson RA, Salford LG, Siesjo BK (1973) Optimal freezing conditions for cerebral metabolites in rats. Journal of neurochemistry 21: 1127-1138.

7. Raghavendra V, Tanga FY, DeLeo JA (2004) Complete Freunds adjuvant-induced peripheral inflammation evokes glial activation and proinflammatory cytokine expression in the CNS. Eur J Neurosci 20: 467-473.

8. Valles SL, Blanco AM, Pascual M, Guerri C (2004) Chronic ethanol treatment enhances inflammatory mediators and cell death in the brain and in astrocytes. Brain Pathol 14: 365-371.

9. Hardin-Pouzet H, Krakowski M, Bourbonniere L, Didier-Bazes M, Tran E, et al. (1997) Glutamate metabolism is down-regulated in astrocytes during experimental allergic encephalomyelitis. Glia 20: 79-85.

10. Beraud E, Viola A, Regaya I, Confort-Gouny S, Siaud P, et al. (2006) Block of neural Kv1.1 potassium channels for neuroinflammatory disease therapy. Ann Neurol 60: 586-596.

11. O'Flaherty JT, Wykle RL (2004) Biochemical interactions of platelet-activating factor with eicosanoids. In: Curtis-Prior P, editor. The Eisocanoids. West Sussex: Wiley.

12. Fonteh AN (2004) An outline of arachidonate remodeling and its biological significance. In: Fonteh AN, Wykle RL, editors. Arachidonate remodeling and inflammation. Basel: Birkhäuser. pp. 1-12.

13. Lampa J, Westman M, Kadetoff D, Agreus AN, Le Maitre E, et al. (2012) Peripheral inflammatory disease associated with centrally activated IL-1 system in humans and mice. Proc Natl Acad Sci U S A.

14. Farooqui AA, Horrocks LA, Farooqui T (2007) Modulation of inflammation in brain: a matter of fat. J Neurochem 101: 577-599.

15. Woelk H, Kanig K (1974) Phospholipid metabolism in experimental allergic encephalomyelitis: activity of brain phospholipase A1 towards specifically labelled glycerophospholipids. J Neurochem 23: 739-743.

16. Marusic S, Leach MW, Pelker JW, Azoitei ML, Uozumi N, et al. (2005) Cytosolic phospholipase A2 alpha-deficient mice are resistant to experimental autoimmune encephalomyelitis. J Exp Med 202: 841-851.

17. Hegen M, Sun L, Uozumi N, Kume K, Goad ME, et al. (2003) Cytosolic phospholipase A2alpha-deficient mice are resistant to collagen-induced arthritis. J Exp Med 197: 1297-1302.

18. Yedgar S, Cohen Y, Shoseyov D (2006) Control of phospholipase A2 activities for the treatment of inflammatory conditions. Biochim Biophys Acta 1761: 1373-1382.

19. Farooqui AA, Ong WY, Horrocks LA (2004) Biochemical aspects of neurodegeneration in human brain: involvement of neural membrane phospholipids and phospholipases A2. Neurochem Res 29: 1961-1977.
